# Dietary DNA Metabarcoding From Animal Fecal Samples

**DOI:** 10.1002/cpz1.70226

**Published:** 2026-01-20

**Authors:** Rachel D. McConnell, Crinan Jarrett, Diogo F. Ferreira, Luke L. Powell, Alma L. S. Quiñones, Davide M. Dominoni, Andreanna J. Welch

**Affiliations:** ^1^ Department of Biosciences Durham University Durham United Kingdom; ^2^ School of Biodiversity, One Health and Veterinary Medicine University of Glasgow Glasgow United Kingdom; ^3^ Biodiversity Initiative Houghton Michigan USA; ^4^ Bird Migration Unit Swiss Ornithological Institute Sempach Switzerland; ^5^ CIBIO‐InBIO, Research Center in Biodiversity and Genetic Resources University of Porto Vairão Portugal; ^6^ BIOPOLIS Program in Genomics, Biodiversity and Land Planning, CIBIO Campus de Vairão Vairão Portugal

**Keywords:** DNA extraction, high‐throughput sequencing, laboratory workflow, metabarcoding, PCR amplification

## Abstract

Fecal DNA metabarcoding is a powerful tool for examining animal diets with unprecedented resolution, offering insights into ecological patterns shaped by trophic interactions. As a result, dietary metabarcoding has become widely applied across ecology, evolution, behavior, and conservation. This article provides a practical guide to the key steps involved in metabarcoding animal fecal samples, from field collection and storage through to laboratory processes, such as DNA extraction, PCR amplification, and sequencing library preparation. It also outlines a bioinformatics workflow using the open‐source QIIME2 platform to filter, error‐correct, and assign taxonomy to dietary DNA sequences. We present key considerations for study design, highlighting potential caveats and limitations to enable researchers to make informed methodological choices. In addition, we offer guidance on the statistical analysis of diet data, including generalized linear models, multivariate analyses, and network analyses. © 2026 The Author(s). Current Protocols published by Wiley Periodicals LLC.

**Basic Protocol 1**: Metabarcoding library preparation of animal fecal dietary DNA for Illumina MiSeq sequencing

**Support Protocol 1**: Making a SpeedBeads solution

**Support Protocol 2**: Calibrating the SpeedBeads solution

**Basic Protocol 2**: QIIME2 bioinformatics workflow for metabarcoded dietary DNA

## INTRODUCTION

Determining the diet of animals is vital to understanding their ecology, trophic relationships, and role within ecosystems. Diet studies have provided novel insights into the interactions that shape ecological communities (Sow et al., 2020), intra‐ and inter‐specific niche specialization (Andriollo et al., 2021), as well as ecosystem services, such as pest control (Kemp et al., 2019) and seed dispersal (Myers et al., 2004). Defining the diet of animals has also been instrumental to understanding how species are adapting to changing landscapes (Burger et al., 2012; Pollock et al., 2017).

A range of techniques exist to identify the composition of an animal's diet. Direct observation of feeding behavior can be useful but is often limited by observer bias and poor visibility of rare, elusive, or nocturnal species (Santana et al., 2011). Morphological analysis of prey remains (Moorman et al., 2007), gut contents (Avanesyan, 2014), or feces (Rytkönen et al., 2019) is labor‐intensive and limited by taxonomic resolution and observer expertise (De Sousa et al., 2019). Morphologically similar prey can be misidentified or only identified to a low resolution (Zeale et al., 2011), while soft‐bodied or digested species may go undetected (Berry et al., 2017). Stable isotope analysis, which compares, for example, isotopic ratios of carbon, nitrogen, and/or sulfur in animal tissues provides dietary information over time but cannot identify prey to the species level when isotope signatures are similar (Traugott et al., 2007; Vander Zanden et al., 2015). Recently, molecular diet analysis has become more widely implemented.

**Figure 1:**
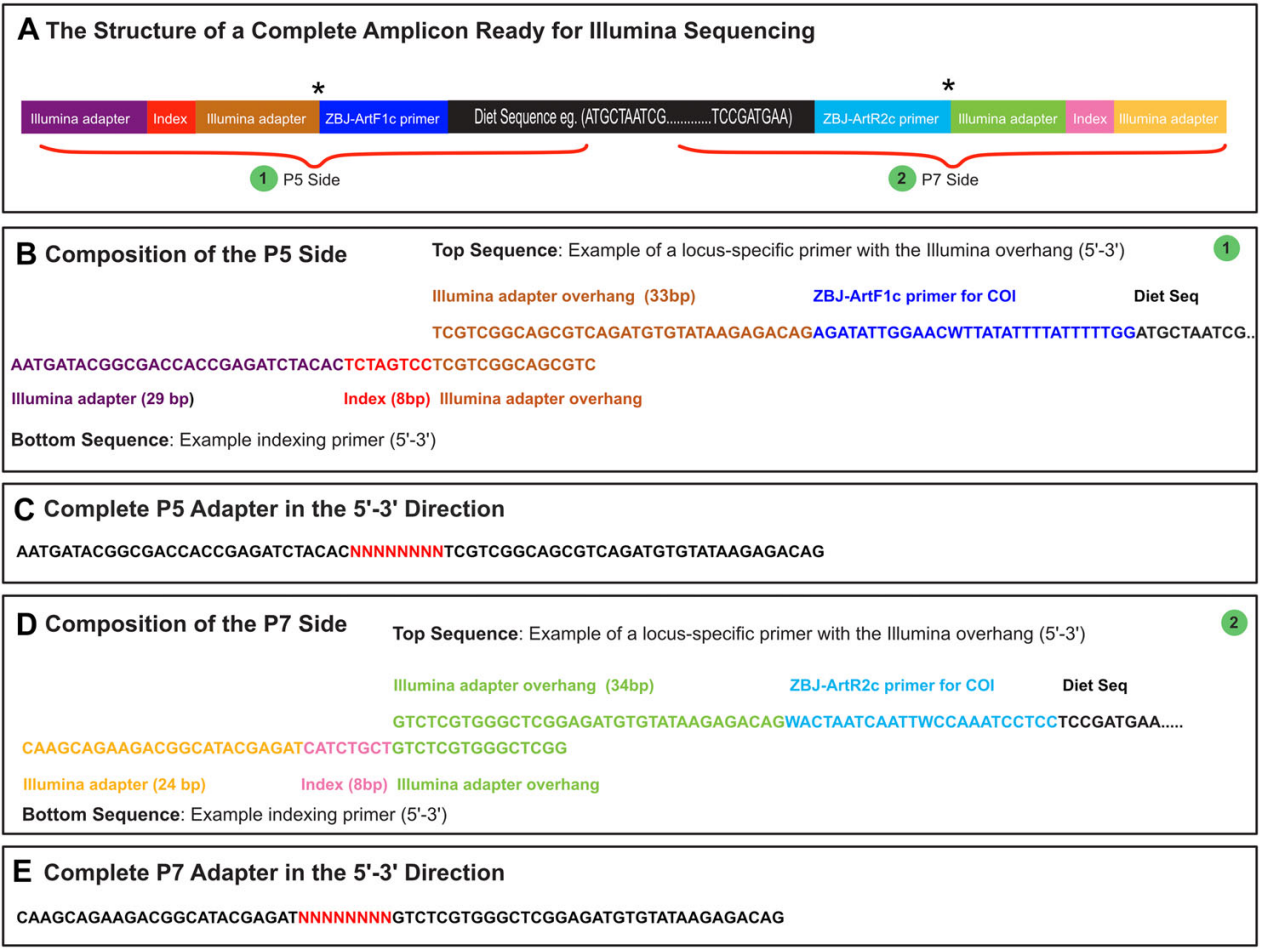
The structure of an amplicon from fecal DNA metabarcoding prepared for Illumina sequencing (following Basic Protocol 1) using the ZBJ primers (Zeale et al., 2011). (**A**) A diagram of a fully prepared molecule demonstrating the location of the PCR primers and Nextera‐style Illumina adapters with unique indices. Optionally, amplicon‐specific tags or barcodes may be utilized (location indicated by asterisks). (**B**) P5 side of an amplicon, showing the locus‐specific primer above and the indexing primer below. (**C**) The complete P5 adapter, where the red N's denote the location of an individual‐specific index sequence. (**D**) P7 side of an amplicon, with the locus‐specific primer above and the indexing primer below. (**E**) The complete P7 adapter, where the red N's denote the location of an individual‐specific index sequence.

Historically, molecular diet analysis began with techniques like antibody detection and protein electrophoresis (Symondson, 2002). The development of PCR (Saiki et al., 1988) and Sanger sequencing (Sanger et al., 1977) enabled the amplification and detection of minuscule quantities of prey DNA from degraded samples (Höss et al., 1992; King et al., 2008). Early approaches used species‐specific primers (Asahida et al., 1997) or universal primers and cloning to resolve mixed prey assemblages (Poinar et al., 1998; Pompanon et al., 2012). A major conceptual advance was the barcode theory (Arnot et al., 1993), later formalized by Hebert et al. (2003) who advocated for a standardized single‐locus barcode. The shift to high‐throughput sequencing (HTS) technologies (Margulies et al., 2005) eliminated the need for cloning and enabled comprehensive dietary studies at unprecedented scale and resolution. One such method is metabarcoding. Metabarcoding works by PCR amplifying a highly variable genomic region, such as cytochrome c oxidase subunit I (COI), using general primers that bind to conserved flanking sequences (Fig. 1A). These amplicons are sequenced using HTS platforms and assigned taxonomy through comparison with reference databases. This approach enables simultaneous analysis of large sample sets and offers semi‐quantitative insights into dietary composition, prey richness, and trophic niche partitioning (Clare et al., 2009; Deagle et al., 2009; Jedlicka et al., 2013).

DNA metabarcoding has emerged as a leading approach, offering a powerful, non‐invasive alternative to characterize diet to a high taxonomic resolution compared to traditional morphological analyses (Johnson et al., 2021; Kartzinel & Pringle, 2020; Nboyine et al., 2019; Sow et al., 2020). Metabarcoding is particularly effective for dietary studies when traditional methods fall short, such as for rare, nocturnal, aerial, or soft‐bodied prey consumers. It is especially valuable in comparative studies where full dietary resolution is not essential, and in projects with large sample sizes (e.g., >100), where it can save substantial time compared to microscopic analysis. It also complements other tools like stable isotope analysis to provide a more complete dietary profile (Hoenig et al., 2022). Feces are often used as a source of degraded prey DNA as it is relatively easy to obtain and minimally invasive (Moran et al., 2019; Wray et al., 2021). Gut contents or regurgitates are also used, though these often contain higher levels of consumer DNA, especially in small‐bodied taxa like insects (Staudacher et al., 2011; Titulaer et al., 2017). Today, diet metabarcoding is applied across taxa, including birds (Jarrett et al., 2020; Moran et al., 2019), mammals (Thuo et al., 2020), fish (Johnson et al., 2021), reptiles (Marques et al., 2022), and invertebrates (Cuff et al., 2021) to examine subjects, such as trophic interactions, dietary shifts, the impacts of urbanization, invasive species, and supplemental feeding (Camp et al., 2020; Jarrett et al., 2020; Shutt et al., 2021). The data obtained are typically the presence/absence or relative read abundance (i.e., sequence abundance) of a taxon, which allows inference of dietary diversity and niche overlap, though it cannot directly provide the absolute biomass consumed (Deagle et al., 2019).

Metabarcoding has several limitations, including PCR bias, incomplete reference databases, and difficulties in quantification. It also requires specialized laboratory protocols and bioinformatics pipelines tailored to each study system (Mathon et al., 2021; Stapleton et al., 2022). This article provides a step‑by‑step guide to diet metabarcoding, from project design and sample collection to laboratory and analytical workflows. These workflows are broadly applicable to other metabarcoding studies, including those investigating microbiome (Prewer et al., 2023) or environmental DNA from soil (Dopheide et al., 2019), water (A. Murray et al., 2025), or air (Roger et al., 2022), but they must be tailored to the specific study system. Basic Protocol 1 outlines laboratory methods for preparing animal diet DNA sequencing libraries for Illumina MiSeq using birds and bats as a case study. The protocol includes: (1) DNA extraction; (2) PCR amplification of target loci; (3) amplicon purification; (4) pooling amplicons from multiple primer sets (optional); (5) indexing PCR; (6) amplicon purification, normalization, and pooling; and (7) sequencing pool validation. Basic Protocol 2 details a QIIME2 (Bolyen et al., 2019) bioinformatics pipeline, using ZBJ (Zeale et al., 2011) amplicons as a case study, and covering adapter and primer removal, error correction, and taxonomic assignment. We provide dietary data from birds and bats in Cameroon, along with example scripts for statistical analyses including generalized linear models (GLMs), multivariate, and network analyses. We discuss both the strengths and limitations of metabarcoding, offering strategies to address common pitfalls.

## STRATEGIC PLANNING

### Suitability of metabarcoding

First, it is essential to assess whether metabarcoding is an appropriate tool for the study question. Several limitations have been thoroughly discussed in the literature (Alberdi et al., 2019; Lamb et al., 2019; Tercel et al., 2021), and in some instances, metabarcoding may be unsuitable. For studies targeting a small number of known species (e.g., an endangered species, key crop pests), diagnostic PCR combined with agarose electrophoresis or qPCR can provide a cheaper and more targeted alternative (Rubbmark et al., 2018). Furthermore, metabarcoding typically offers only a short‐term dietary snapshot. Capturing long‐term trends (e.g., across an entire breeding season) would require large numbers of samples, which may become logistically unmanageable, making approaches such as stable isotope analysis more practical. Taxonomic suitability of primers and reference databases are also important considerations. Several studies have been conducted on amplification success of universal primers using mock communities, i.e., DNA mixtures from numerous known species (Elbrecht et al., 2019; Jusino et al., 2019). Bioinformatics programs can perform in silico PCR using reference databases such as BOLD (Tournayre et al., 2020) and GenBank (Clarke et al., 2014) to evaluate whether diet item sequences are represented in the database and are likely to be successfully amplified. Taxonomic resolution may also vary geographically, as global reference databases are often biased towards North American and European taxa (Sow et al., 2020; Subrata et al., 2021). Consequently, additional effort may be required to augment existing databases or to develop a comprehensive local reference library. Shotgun metagenomics can circumvent some PCR biases when DNA concentrations are high (Srivathsan et al., 2015), but its costs remain substantially higher than metabarcoding (Cordone et al., 2022; Massey et al., 2021) and issues with reference databases still exist.

In species closely related to their prey (e.g., spiders eating insects or mammals eating other mammals), host DNA often dominates during amplification, masking prey sequences (Vestheim & Jarman, 2008). To address this, strategies should be identified prior to lab procedures such as designing prey‐specific primers or employing host DNA‐blocking primers (Lafage et al., 2020; Piñol et al., 2014, 2015). These primers should be tested and the laboratory procedures optimized as suppression of host DNA is difficult, especially when predator and prey are phylogenetically close (O'Rorke et al., 2012). Metabarcoding can produce sequences from diet items consumed by the prey of the study species: this is an issue known as secondary consumption (Sheppard et al., 2005). Secondary consumption could lead to the incorrect conclusion that a species is omnivorous or exaggerate the importance of items not directly in its diet (Da Silva et al., 2019). Diet metabarcoding also does not provide information on the life stage of the diet items consumed, and this may have implications for answering the study question. Use of multiple methods may be necessary. For example, feeding observations could be used alongside DNA metabarcoding to provide additional information, such as feeding rate and prey characteristics to understand feeding ecology more comprehensively (Jarrett et al., 2020).

### Sample collection

Several sampling design considerations must be addressed before starting a metabarcoding study. The consumer's life stage can influence the proportion of dietary and consumer DNA in feces (McInnes, Alderman, Lea, et al., 2017), and sample timing should reflect feeding biology and seasonal prey fluctuations (Gil et al., 2020). For example, infrequently feeding carnivores may produce feces containing dietary information over an extended period or feces dominated by their own DNA if they have not fed recently (Shehzad et al., 2012; Thuo et al., 2020). In contrast, frequently feeding songbirds generate samples that capture short term dietary information (Jarrett et al., 2020; Oehm et al., 2011). As diet can vary seasonally and even daily, sampling must be carefully timed to align with the study's objectives (Humphries et al., 2017).

Selecting the appropriate sampling unit is also crucial. Individual samples may be required when linking diet to traits like breeding success (Jarrett et al., 2020) whereas pooled samples from multiple individuals may suffice for broader dietary questions (Ford et al., 2016). However, pooling samples can obscure rare diet items; Mata et al. (2019) recommend extracting DNA from individual samples when detecting uncommon prey is important. Practical considerations, such as processing costs, feasibility of collecting individual samples, and available laboratory time will also influence sampling decisions (Alberdi et al., 2019). It is advisable to collect more samples than immediately required, as surplus samples can be used for primer optimization and in further studies when funding permits.

Feces can be collected directly from animals during capture, which offers the benefit of associating diet with individual‐level morphological and life‐history data and reduces environmental contamination (Gil et al., 2020; Johnson et al., 2021; Kaunisto et al., 2017). Small animals can be placed in clean containers or bleached cloth bags to facilitate fecal collection (Aizpurua et al., 2018; Gil et al., 2020; Kaunisto et al., 2017). Alternatively, fecal sacs can be collected directly from nestlings by gently massaging the abdomen and positioning them over a tube (Jarrett et al., 2020; Trevelline et al., 2018). Alternatively, feces can be collected non‐invasively from habitats, commonly used sites, or by tracking animals until defecation (Andriollo et al., 2021; Kartzinel et al., 2019; Lefort et al., 2020; Shutt et al., 2021; Thuo et al., 2020). This is particularly useful when studying rare or elusive species or where invasive methods are not appropriate. Detection dogs have also been used to locate feces of specific species (Ayres et al., 2012; Schmidt et al., 2018). When the sample origin is unknown, host DNA can be amplified to confirm taxonomy (Kartzinel et al., 2015). Environmental samples are more susceptible to contamination or degradation, with substrate affecting prey DNA recovery. Oehm et al. (2011) showed that fewer prey were detected in carrion crow feces collected from soil compared to vegetation or plastic tubes, although prey was still detectable after 5 days of sun and rain exposure. Fecal samples from larger animals may be collected in paper bags (Thuo et al., 2020) or plastic zipper storage bags. For terrestrial mammals, the freshest feces are typically sampled from the center of the scat to avoid contamination (Christopherson et al., 2019; Kartzinel et al., 2015; Massey et al., 2021), and freshness can be assessed based on visual cues and site visits (Massey et al., 2021). Negative controls from nearby, feces‐free substrates can help detect environmental contamination.

*NOTE*: All protocols involving animals must be reviewed and approved by the appropriate Animal Care and Use Committee and must follow regulations for the care and use of laboratory animals.

### Sample storage

Feces must be preserved immediately to prevent DNA degradation. This is especially important for taxa like birds, amphibians, and reptiles that excrete both feces and uric acid through the cloaca, as uric acid can degrade DNA and inhibit PCR amplification (Huggett et al., 2008; Khan et al., 1991). Preservation options include immediate freezing, buffer solutions, or drying (Ando et al., 2020). The preservation method affects downstream DNA extraction and should be selected carefully. For instance, ethanol must be removed prior to extraction as it inhibits PCR. Freezing is effective and supports multiple analyses (Kartzinel et al., 2019; Massey et al., 2021), but may require liquid nitrogen or dry ice, posing logistical challenges. Buffers like Longmire's with 2% SDS are effective for birds and bats, even during temporary storage without refrigeration, and facilitate international sample transfer (Longmire et al., 1988). Over 2000 bird and bat samples preserved this way showed 70% to 95% amplification success (unpublished data). Ethanol is widely used due to its pathogen‐neutralizing properties, and Trevelline et al. (2016) demonstrated successful DNA recovery from bird feces stored at room temperature in ethanol for 3 months. However, it presents drawbacks including evaporation, flammability, shipping constraints, and inhibition of subsequent PCR. Alternatives like RNAlater are used less frequently (Ando et al., 2020). Silica gel with cool storage (4° to 8°C) has worked well for preserving mammal feces (Arrizabalaga‐Escudero et al., 2019; Janečka et al., 2008; Mata et al., 2019). Regardless of short‐term preservation, long‐term storage at –80° or –20°C is preferred (Crisol‐Martínez et al., 2016; Gerwing et al., 2016; Jarrett et al., 2020).

### Choosing a library preparation approach

While early metabarcoding studies used a variety of sequencing platforms available at the time (e.g., 454 sequencing), most recent metabarcoding studies have utilized Illumina sequencing technology, which is compatible with the protocols detailed below. Some authors have explored the use of other methods, such as long‐read sequencing using Oxford Nanopore technologies (Doorenspleet et al., 2025; Huggins et al., 2024). Long‐read sequencing offers several advantages, including higher taxonomic resolution, improved discrimination of closely related species, and enhanced taxon detection (Doorenspleet et al., 2025). However, it also presents disadvantages, such as higher costs per base, elevated sequencing error rates, increased formation of chimeric sequences, and its incompatibility with most bioinformatic tools that were originally designed for short reads, although new tools are being developed (Heeger et al., 2018; Latz et al., 2022). Furthermore, longer DNA fragments may be less abundant in the environment due to degradation, meaning that long‐read sequencing can sometimes detect fewer taxa than traditional short‐read metabarcoding (Doorenspleet et al., 2025).

Prior to beginning a study using Illumina sequencing technology, it is important to select an approach for adding sequencing adapters to amplicons (i.e., building amplicons into Illumina sequencing libraries), as in many cases this involves appending a portion of the sequencing adapter directly to the locus‐specific PCR primers. See Bohmann et al. (2022) for a detailed review of the options and their advantages and disadvantages. All involve adding sample‐specific indices, tags, or barcodes necessary for multiplexing (i.e., combining) samples into the same sequencing run to reduce costs. The terms index, barcode, and tag can be confusing for those new to metabarcoding, and they are often used inconsistently by practitioners. Here, an index refers to a short string of nucleotides (often 6 to 8 bp) situated within the Illumina adapter (Fig. 1A), which are read during specific index reads by the sequencing platform. Often data received from the sequencing facility will have been demultiplexed such that sequences associated with each unique pair of indices will already be sorted into separate files. These index sequences are generally not found within the resulting DNA sequences. Here, tags and barcodes are used synonymously and refer to short strings of nucleotides added outside of the Illumina adapter, directly next to the locus‐specific PCR primer. Because these are outside the Illumina adapter, they are sequenced within the forward and reverse reads, the same as the PCR primers and dietary sequence. Typically, data received from the sequencing facility will not have been demultiplexed based on the tag sequences, so data files may still contain sequences from multiple samples, thus requiring an extra bioinformatic demultiplexing step to sort them appropriately.

Briefly, there are three general approaches to library preparation. First, it is possible to construct full Illumina libraries from PCR products using multiple enzymatic reactions (e.g., ligation of adapters), though this will be relatively expensive and time consuming (Clarke et al., 2014). Second, it is possible to append the entire Illumina adapter, including individual‐specific index and/or barcode sequences, to the locus‐specific primer (fusion primers, Elbrecht & Leese, 2015). This is relatively time efficient; however, this approach becomes expensive if many samples are to be pooled into the same sequencing lane, as each sample will require its own unique combination of long primers (∼80 bp or more).

The final general approach is a two‐step PCR approach, either with or without tagging. Here, partial Illumina adapters are appended onto the locus‐specific PCR primers (Fig. 1, Taberlet et al., 2018). After the first round of PCR, this overhanging region becomes the template for a second PCR with a small number of cycles that uses complementary “indexing primers” to extend the Illumina adapter to full length and add a sample‐specific index. Tags can be introduced in the first PCR round by appending a small number of nucleotides (e.g., 6 to 8 bp) between the locus‐specific primer and the partial Illumina adapter overhang (Fig. 1). See the Supplemental Information of Vamos et al. (2017) for possible tag combinations.

The major benefits of tagging include being able to bioinformatically detect cross‐contamination occurring at early stages of the laboratory work pipeline, ability to track diet inference from different technical PCR replicates, and the ability to detect “tag jumps” or “index hops”, when DNA molecules acquire the wrong index or tag sequence and can subsequently be mis‐assigned to samples during bioinformatics analyses (Schnell et al., 2015). Pooling of multiple tagged amplicons within the same set of indices can also be cost effective for sequencing on the recent Illumina platforms, where many external sequencing companies require a large amount of data per index combination (e.g., 0.5 GB or ∼1.6 million 150 bp–paired sequences) because of issues related to index‐hopping. Major drawbacks of this tagged approach include increased cost (more primer pairs are required overall) and additional complexity during PCR set up as each reaction requires a different primer. If amplification of pooled tagged amplicons is conducted, higher levels of tag jumping could occur. Here we use the untagged approach because of its relative simplicity, ease for new practitioners, and lower starting costs. Following this protocol, amplicons can be cheaply sequenced on an Illumina MiSeq platform. The protocols presented in this article are compatible with both the tagged and untagged two‐step PCR approaches; however, different PCR primers would need to be ordered for each.

## Basic Protocol 1 METABARCODING LIBRARY PREPARATION OF ANIMAL FECAL DIETARY DNA FOR ILLUMINA MISEQ SEQUENCING

**Figure 2:**
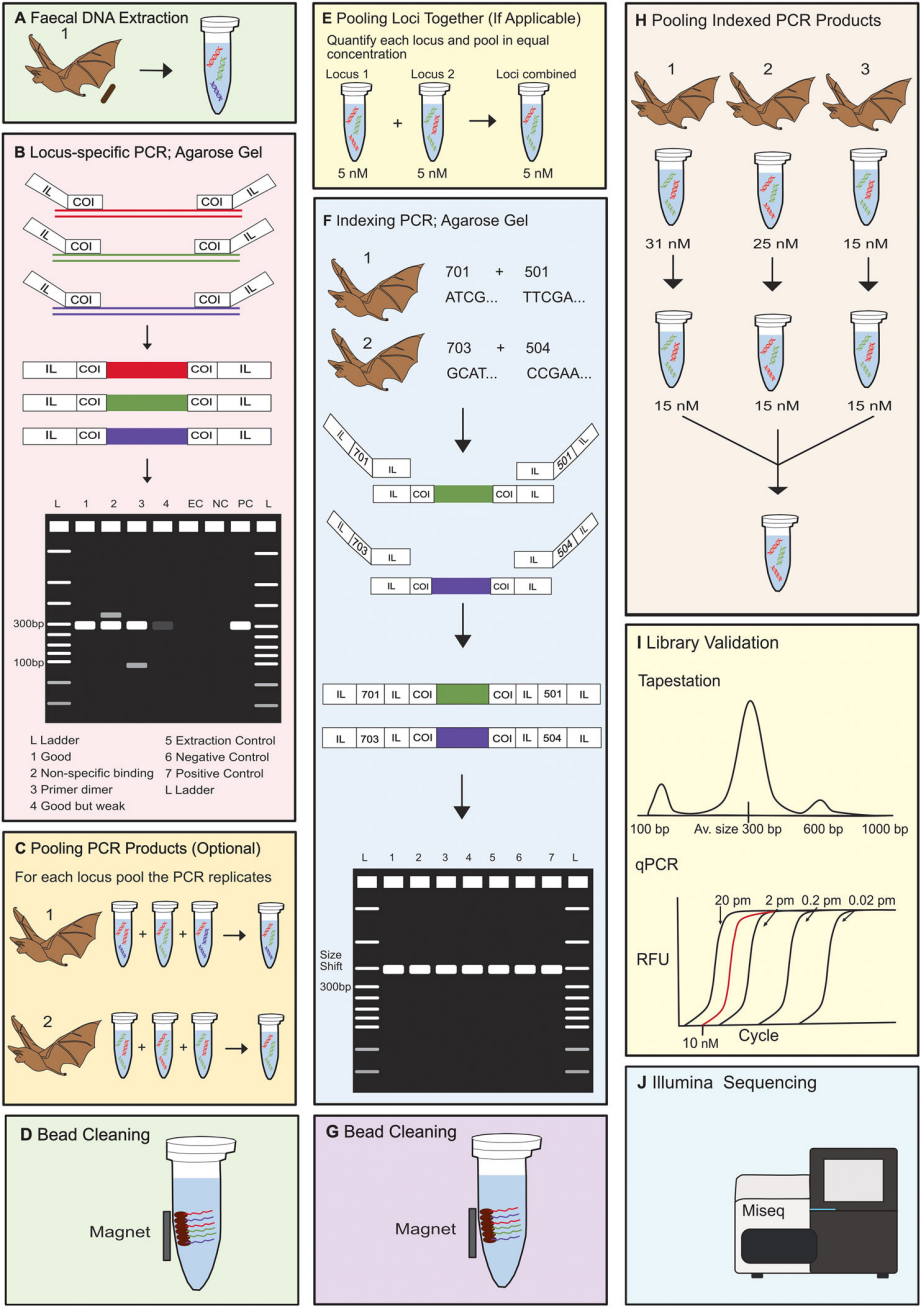
Overview of a metabarcoding laboratory pipeline showing: (**A**) fecal DNA extraction; (**B**) locus‐specific PCR (targeting the COI locus) with Illumina overhangs (IL) appended to each primer, followed by gel electrophoresis to check amplification success; (**C**) pooling of technical PCR replicates for each locus separately for each individual (optional); (**D**) bead cleaning the pooled amplicons; (**E**) pooling the combined amplicons of each locus together in equal concentration for each individual (if applicable); (**F**) indexing the pooled clean amplicons and checking for size shift using gel electrophoresis; (**G**) bead cleaning the indexed amplicons; (**H**) equalizing the concentration of the indexed amplicons where possible followed by the pooling of all samples into a single tube; (**I**) library validation using a Tapestation instrument to check the quality of the library (above) and qPCR to determine the concentration (below); and (**J**) sequencing on an Illumina platform.

The aim of this protocol is to prepare fecal dietary DNA for metabarcoding by employing an untagged two‐step PCR approach. We use bird and bat fecal samples as an example, but steps apply to other taxa as well. This protocol involves several steps starting with the extraction of dietary DNA using the Omega EZNA tissue extraction kit, locus‐specific PCR amplification using a Qiagen Multiplex PCR kit, the pooling of PCR replicates, amplicon purification with magnetic SpeedBeads, an indexing PCR and amplicon purification, normalization, and pooling followed by sequencing pool validation (Fig. 2). The protocols described here include steps we generally follow, as we have found them to be both time and cost efficient. Undoubtedly, other researchers will have different preferences for some steps (e.g., alternative methods for adding sequencing adapters, conducting PCR product clean‐up, etc.) that are equally valid and also produce good results (Elbrecht & Leese, 2015; Oehm et al., 2011)

*CAUTION*: An overarching consideration for all laboratory steps is the prevention of contamination (Alberdi et al., 2019; Thomsen & Willerslev, 2015). This is a particular issue for fecal diet metabarcoding samples because target diet DNA in feces is generally degraded and present in relatively low abundance, and universal primers will readily amplify organisms from a very broad taxonomic range. Thus, contamination may be preferentially amplified during PCR. To minimize contamination, we highly advise having separate spaces to carry out the pre‐ and post‐PCR procedures (see Troubleshooting). Metabarcoding is sensitive enough to identify DNA from the air (Banchi et al., 2020; Lynggaard et al., 2022), so shared lab spaces should be used only with extreme caution. We recommend cleaning all workspaces and pipettes with diluted bleach (i.e., 20% bleach solution) when starting a new task and using filter tips. All consumables used, such as tubes and glassware, should either be bought sterilized or autoclaved. It is also very important to use controls (Alberdi et al., 2019; Thomsen & Willerslev, 2015). Negative controls in the DNA extraction step (hereafter referred to as extraction controls) and during PCR (hereafter referred to as PCR negative controls) help identify if the samples or reagents become contaminated (Taberlet et al., 2018). Positive controls, DNA of high‐quality specimens not present in the study, should also be used to confirm that the PCR reaction was successful.

### Materials

Fecal sample
Longmire buffer (see Reagents and Solutions)
Sodium hypochlorite (bleach) (Thermo Fisher Scientific, cat. no. 11438842)
Omega EZNA tissue extraction kit (Omega, cat. no. D3396‐02)
100% ethanol (Sigma‐Aldrich, cat. no. E7023)
100% isopropanol (Sigma‐Aldrich, cat. no. 190764)
Gordon buffer (see Reagents and Solutions)
Locus‐specific DNA primers (e.g., ZBJ, Fwh2, trnl, etc.) with Illumina adapters appended (see Critical Parameters)
Positive DNA control (see step 37 for more details)
Pre‐PCR hood
Qiagen Multiplex PCR kit (Qiagen, cat. no. 206143)
Ice
10 mM Tris·HCl pH 8.0 (Sigma‐Aldrich, cat. no. 93363)
H_2_O, nuclease‐free (New England Biolabs, cat. no. B1500S)
Gel electrophoresis reagents:
  Agarose (Sigma‐Aldrich, cat. no. A9539)
  Ethidium bromide (Thermo Fisher Scientific, cat. no. 15585011)
  Low molecular weight DNA ladder (New England Biolabs, cat. no. N3233L)
  Tris‐borate‐EDTA (TBE) buffer (Thermo Fisher Scientific, cat. no. B52)
Agencourt AMPure XP beads (Beckman Coulter, cat. no. A63880) or home‐made Sera‐Mag SpeedBeads (Cytiva, cat. no. 6515‐2105‐050250; see Support Protocols 1 and 2)
Qubit dsDNA high sensitivity (Thermo Fisher Scientific, cat. no. Q33230) and broad range (Thermo Fisher Scientific, cat. no. Q33265) assay kits
Qubit assay tubes (Thermo Fisher Scientific, cat. no. Q32856)
Unique indexing primers (see step 72)
KAPA HiFi HotStart ReadyMix (Roche, cat. no. 7958927001)

2‐, 20‐, 200‐, and 1000‐µl pipettes and filter tips (multichannel pipettes are optional)
50‐ and 100‐ml serological pipettes and pipette pump
50‐ml centrifuge tubes
Water bath
2‐ml screw‐cap tubes, sterile
0.5‐mm silica‐zirconia beads (Biospec, cat. no. 11079105z)
Spatula with small spoon, sterile or cleaned with 50% bleach
≥2 sterile tweezers, long and thin enough to reach the bottom of the 2‐ml screwcap tubes
Tissue paper
Bunsen burner
Sterile cleaning wipes (since tissue in bleach sticks to gloves)
Balance with ≥0.001 g precision
Square weigh boats (44 × 44–mm or larger)
Qiagen Tissuelyser II or similar for vigorous sample homogenization
Centrifuge (e.g., Eppendorf, cat. no. 5415D)
Dry bath
2‐ml snap‐cap tubes, sterile
Vortex
1.5‐ml DNA LoBind tubes (Eppendorf, cat. no. 003010851)
Aluminum foil
0.2‐ml PCR tubes
96‐well PCR cooler plate (e.g., Eppendorf, cat. no. EP3881000031)
Cool polystyrene box
PCR thermocycler
Gel electrophoresis system
Magnetic rack, e.g., MAGBIO MyMag 96× magnetic plate
Reagent reservoirs

Other optional reagents and equipment include: TapeStation system and computer (Agilent, cat. no. 4150); TapeStation High Sensitivity D1000 ScreenTape (Agilent, cat. no. 5067‐5584); TapeStation High Sensitivity D1000 reagents (Agilent, cat. no. 5067‐5585); Agilent TapeStation High Sensitivity D1000 ladder (Agilent, cat. no. 5067‐5587); CFX Duet Real‐Time PCR system (Bio‐Rad); 0.2‐ml 8‐Tube PCR strips and caps, optically clear (Bio‐Rad, cat. no. TBC0802); and library quantification kits, e.g., Complete Kit Universal (KAPA, cat. no. KK4824)

#### Section 1: DNA extraction from feces

This section describes the steps to extract dietary DNA from bird and bat fecal samples preserved in Longmire buffer using a column‐based approach. See Critical Parameters section for information on different extraction techniques. The extraction process spans 2 days and yields ∼50 µl DNA per sample.

1 Clean the workbench and outer surfaces of the pipettes with 20% bleach.
  If possible, use a pre‐PCR hood as well.
2 Prepare the buffers of the Omega EZNA tissue extraction kit by adding ethanol or isopropanol as directed using the serological pipettes and pipette pump.
3 Take 23 fecal samples out of the freezer.
  We recommend starting with a smaller batch the first time. If samples have been stored in ethanol, the ethanol must be removed prior to continuing the protocol.
4 Transfer 16 ml of Gordon buffer, which includes extra for pipetting error, into a 50‐ml centrifuge tube using a seriological pipette and pump and place it in a water bath at 56°C.
5 Label 24 2‐ml sterile screw‐cap tubes. These are used for weighing out the feces and includes a tube for the extraction control. The labels often rub off during the protocol so it is beneficial to label the side and lid of each tube.
6 Add 0.5 g of 0.5‐mm silica‐zirconia beads to each screw‐cap tube using a sterilized spatula, ideally with a small spoon on the end.
7 Sterilize the tweezers with 50% bleach, blot on tissue paper, then place in ethanol and heat over a Bunsen burner to dry.
  Take extra care during this step to avoid getting ethanol on your gloves, as this poses a serious fire risk when working near an open flame. Ensure all ethanol has evaporated before using as it can inhibit DNA amplification.
8 Cover a wipe with bleach for cleaning gloves between samples.
9 Place 60 to 80 mg (0.06 g, wet weight) of the fecal sample into a tube with the silica‐zirconia beads.
  See Critical Parameters for further details on selecting an appropriate sample weight and sampling strategy for your study system. A suggested approach for different sample types is below. If insufficient feces are available, and the samples have been stored in a suitable buffer (like Longmire buffer with 2% SDS; NOT ethanol), you can boost the sample weight by adding the sample's buffer to achieve a minimum of 60 mg, though results may still be poor.
  a. For chick fecal sac samples, use the tweezers to remove the fecal sac from each tube and place in a small weigh boat. Use 2 tweezers to dissect the blackish feces away from the sac and white uric acid portion (the latter can be discarded).
  b. For other bird fecal samples, use a small spatula to remove the blackish feces from the sample tube and place it in the tube with the silica‐zirconia beads. Avoid white uric acid portion.
  c. For bats, use a small spatula to remove the fecal sample from the tube. A single fecal pellet may not reach the ideal weight, and you may decide to add more (Mata et al., 2019).
  d. Extraction control. Do not add a sample to the extraction control tube. This will allow detection of contaminants in the extraction process.
    We recommend removing uric acid as it inhibits DNA amplification (Davis, 1927); however, metabarcoding studies on reptiles and amphibian feces have been successful without removing the urine component from the sample (Gil et al., 2020; Marques et al., 2022). It is beneficial to place the fecal sample at least 1/3 of the way down the tube so that it does not get stuck up in the lid or at the sides of the tube. If the fecal sample is insufficient and you need to pipette the buffer along with parts of the feces, use a 1000‐µl filter tip and cut off the very end of the tip to prevent the feces from getting stuck inside.
10 Wipe gloves with bleach and sterilize the working surface, spatula and tweezers with beach, ethanol, and a Bunsen burner between each sample. Repeat step 9 for each sample.
11 Remove the Gordon's buffer from the water bath and distribute 650 µl into each tube.
12 Place the tubes in a Qiagen Tissuelyser II for 5 min at 25 Hz. Change the orientation of the tubes and place them in the Tissuelyser for a further 5 min at 25 Hz.
  We have compared simply vortexing to using the Tissuelyser, or similar product, and found that more vigorous sample disruption leads to dramatic increases in success.
13 Centrifuge the samples for 1 min at 9240 × *g*, room temperature, to bring the feces to the bottom of the tube.
14 Leave all tubes in an oven or water bath at 56°C for at least 20 hr. Shake or mix occasionally.
15 The next day clean the working space and material with 20% bleach as well as the outer surfaces of the pipettes.
16 Make a 1.3‐ml aliquot of elution buffer from the kit and place this in a water bath or oven at 56°C.
17 Turn on a dry bath or another water bath and set it to 70°C.
18 Label 2‐ml snap‐cap tubes with the sample numbers.
  The labels often rub off during the protocol, so it is beneficial to label the tubes on the lid and the side as a precaution.
19 Prepare aliquots of buffers from the DNA extraction kit, using the serological pipette, pump and 50‐ml centrifuge tubes or 2‐ml snap cap tubes as appropriate, to prevent accidental contamination of the kit stocks.
  a. Make a 600‐µl aliquot of OB Protease.
  b. Make a 4.8‐ml aliquot of BL buffer.
  c. Make a 7.2‐ml aliquot of ethanol.
  d. Make a 12‐ml aliquot of HBC buffer.
  e. Make a 33.6‐ml aliquot of DNA wash buffer.
    The aliquot volumes provided are the minimum amount needed. To allow for pipetting error it is useful to measure out ∼10% extra of what is needed.
20 Distribute 25 µl OB Protease to each of the 2‐ml snap‐cap tubes.
  It is crucial to check after each step that the reagent has indeed been added to all the tubes. To remain organized, it is useful to move each tube once the necessary reagents have been added. Double check that tubes have the same volume of liquid before moving on to the next step.
21 Remove the samples from the water bath and centrifuge 1 min at 9240 × *g*, room temperature. Transfer up to 500 µl supernatant to the 2‐ml snap‐tubes with OB Protease avoiding any sediments and the white membrane on the surface (this forms if the sample has high levels of uric acid). Discard the tube with the beads and any remaining supernatant.
22 Add 200 µl BL buffer to each tube with the supernatant. Vortex at maximum speed for 15 s and centrifuge 30 s at 2000 × *g*, room temperature.
  The BL buffer is quite viscous so it should be pipetted slowly to ensure the correct amount is measured. A new tip should be used for each tube as a little BL buffer is prone to remain in the tip.
23 Place the samples in the 70°C dry bath for 45 min. Use this time to label the columns, collection tubes, and the 1.5‐ml LoBind tubes with the sample numbers and set to one side.
  The LoBind tubes are used for the final elution. Standard 1.5‐ml centrifuge tubes can also be used here if necessary.
24 Centrifuge the samples 30 s at 9240 × *g*, room temperature, to remove condensation.
25 Add 300 µl of 100% ethanol and vortex at maximum speed for 20 s. Centrifuge samples 30 s at 2000 × *g*, room temperature.
  This is a safe place to stop if needed. You can put samples in the freezer (covered entirely in aluminum foil to avoid contamination). If stopping, when restarting the protocol allow samples to come to room temperature.
26 Transfer up to 600 µl of supernatant to the column with a collector tube. Centrifuge 1 min at 9240 × *g*, room temperature. With one hand take the column out of the collector tube and hold it while emptying the collector tube with the other hand. Place the column inside the empty collector tube.
27 Repeat step 26 if there is remaining supernatant.
28 Add 500 µl HBC buffer to the column. Centrifuge 1 min at 9240 × *g*, room temperature, and then empty the collection tube.
29 Add 700 µl DNA wash buffer to the column. Centrifuge 1 min at 9240 × *g*, room temperature, and then empty the collection tube.
30 Repeat step 29.
31 Centrifuge 2 min at 16,110 × *g*, room temperature, to completely dry the membrane.
32 Transfer the column to the corresponding 1.5‐ml labeled LoBind tube. Add 50 µl elution buffer to each column and incubate at room temperature for 5 min. Centrifuge 1 min at 9240 × *g*, room temperature.
33 Transfer the elute from the bottom of the 1.5‐ml LoBind tube back into the same column. Incubate at room temperature for 5 min. Centrifuge 1 min at 16,110 × *g*, room temperature.
  Many kits suggest a relatively large elution volume (e.g., 200 µl of elution buffer once or even twice). Because diet DNA is relatively degraded and in low concentrations, we suggest reducing this to the minimum amount possible. Using the same elution buffer twice concentrates the DNA by doing a second elution but not increasing the overall volume. We have also extended the incubation times during elution to maximize the DNA yield.
34 The extracted DNA will now be contained in the elution buffer in the LoBind tubes. Discard the column.
35 Store the extracted DNA in the freezer.

#### Section 2: Locus‐specific PCR

This section describes the locus‐specific amplification of extracted dietary DNA (Fig. 2B). It produces 15 µl amplicon for each sample. This PCR should ideally be done after each set of extractions to verify that the reagents have not become contaminated. Typically, PCR is repeated until 3 successful amplifications are obtained for each sample. As considerable disparity can occur between PCR replicates, incorporating a greater number provides a more representative account of a species’ diet (Alberdi et al., 2018; Forin‐Wiart et al., 2018). However, the costs and the trade‐offs of incorporating more replicates (e.g., inclusion of fewer individuals in the study) should also be considered (Alberdi et al., 2019).

36 Select appropriate locus‐specific primers for your study.
  Several papers have compared taxonomic breadth of primer sets (Tournayre et al., 2020; Vamos et al., 2017). See Critical Parameters for more details.
37 Prepare a positive DNA control for the PCR. Use a high‐quality, PCR‐positive sample from a species known to amplify with the chosen primers but absent from your study system.
38 Set up the PCR in a dedicated pre‐PCR laboratory and hood. The bench and outer surfaces of the pipettes should be cleaned with 20% bleach. Filter tips should be used at this stage, and all plastics should be sterile.
39 Make a spreadsheet to keep a record of the PCR information. Assign each PCR event a unique ID so that products can be identified for use in subsequent steps. Assign each sample, the extraction control, the positive PCR control, and the negative PCR control a position in the PCR. Label the PCR tubes on the top and sides and place in a 96‐well PCR cooler plate.
  We have found that the best PCR tubes are those with individually attached caps, which are helpful to prevent contamination. Strip tubes with strip lids are likely more prone to contamination, as the entire strip must be removed at once and set down rather than opening tubes individually. It is also more difficult to keep track of what has been added to each tube when the lids cannot be opened one at a time. It is possible to conduct PCR in plates, though these are more prone to contamination and pipetting errors as well.
40 Place the DNA samples, primers, and Multiplex PCR Master Mix on ice in a polystyrene box to keep them cool.
  Primers should be prepared to a concentration of 10 µM using 10 mM Tris·HCl pH 8 before use.
41 Make a master mix for the required number of samples, including controls, and 10% extra for pipetting error. The master mix will contain dH_2_O, Qiagen Multiplex PCR Master Mix, 10 µM forward primer, and 10 µM reverse primer according to the protocol below (see Table 1).
  Note that if using tags, primers cannot be added to the master mix and should be added to each PCR tube directly. Mix all reagents thoroughly prior to use. Avoid vortexing Qiagen Multiplex PCR Mastermix and DNA. Instead mix gently by hand and short spin to collect the reagent at the bottom of the tube.

**Table 1:** Master Mix Protocol for Locus‐Specific Amplification PCR

Protocol	Reaction (µl)	Master mix (µl)
dH_2_O	3.10	3.10 × *n* samples
Qiagen Multiplex PCR master mix	7.50	7.50 × *n* samples
Forward primer (10 µM)	0.2	0.2 × *n* samples
Reverse primer (10 µM)	0.2	0.2× *n* samples

42 Add 11 µl master mix into each labeled PCR tube.
43 Add 4 µl DNA to the PCR sample tube. Add 4 µl of the extraction control to the corresponding PCR tube and add 4 µl nuclease‐free water to the negative control. In the post‐PCR lab, add 1 µl DNA from your pre‐made positive control.
  We have found that adding between 3 and 5 µl of DNA works well, but you may need to adjust depending on your samples. If more or less DNA is added, change the volume of water so that the final volume remains at 15 µl.
44 Mix the PCR tubes gently by hand and centrifuge 30 s at 9240 × *g*, room temperature.
45 Place the PCR tubes into the thermocycler and run the desired program (e.g., see Table 2). Note that increasing the number of cycles will likely give increased amplification success but may also increase the number of PCR artefacts and polymerase errors.

**Table 2:** Example Thermocycler Program for PCR Using QIAGEN Multiplex PCR Master Mix

Process	Temperature (°C)	Time (min:s)	
Polymerase activation	95	15:00	
Denaturation	95	0:30	×35 cycles of denaturation, annealing, and extension
Annealing	46‐52*^a^*	1:00	
Extension	72	0:30	
Final extension	72	10:00	
Holding temperature	15	Forever	

^*a*^ The annealing temperature should be adjusted based on the primers used.

46 Conduct an agarose gel electrophoresis of 5 µl of the sample to assess amplification success and examine the controls for signs of contamination.
  For diet samples a 2% gel often works well. Use an appropriately sized ladder to check the sizes of the bands obtained (Fig. 2B), such as a low molecular weight DNA ladder. If a portion of the Illumina adapters has been appended to the primers, one should ideally expect to see a single band, slightly larger than the size initially described for the PCR product in the literature. However, if using ITS primers, multiple bands may be present due to the wide size variation of this locus among taxa. Amplification from degraded DNA can be weak, but visible bands should produce some sequences. In our experience, mammal samples tend to have higher amplification success than birds, and amplification success often ranges from 60% to 95%. The positive control should have a band of the expected size. The negative and extraction controls should not have a band, though sometimes they do produce primer‐dimer bands of ∼100 bp.
47 Repeat this procedure until you have at least 3 good PCR replicates for each sample.
48 Optional step.
  Technical PCR replicates from the same primer set can be pooled at this point to save time and cost during subsequent steps (Fig. 2C). We do not recommend pooling amplicons from different primer sets at this point, though. Concentrations can vary dramatically between amplicons produced with different primers, even when starting from the same DNA template concentration and even if the intensity of bands visualized on an agarose gel appear similar. We recommend purifying amplicons first, to remove any artefacts (e.g., residual primers, dNTPs), and then assessing concentration using a fluorescent based kit before pooling amplicons from multiple loci (see Section 4 below). If data on composition of technical replicates is required, ensure that amplicons have been tagged prior to pooling. Note, however, tag jumping may occur when amplifying pools of uniquely tagged molecules during indexing (section five).
  a. Identify the desired final volume for pooling that is consistent among samples (as required for bead cleaning, e.g. 15 µl, see Section 3 below). For example, if 3 successful replicate PCRs were obtained then pool 5 µl from each; if only 2 successful replicates were obtained, pool 7.5 µl from each.
  b. Label a new set of PCR tubes and PCR plate for the pooled samples.
  c. Pool the replicates into one PCR tube per locus and sample, changing the pipette tip each time. This requires substantial organization and concentration.

#### Section 3: Amplicon purification

In this section, amplicons are purified using a magnetic speedbead approach to remove any remaining primers and primer‐dimer artefacts to prepare the samples for the indexing PCR (Fig. 2D). The protocol can be conducted using multichannel pipettes to increase efficiency. Pre‐made bead solutions can be purchased (e.g., Agencourt AMPure XP beads), but it is also possible to make a bead solution, used here, at substantially reduced costs (see Support Protocol 1).

During bead clean‑up, magnetic beads preferentially bind larger DNA fragments first, with size selection controlled by adjusting bead concentration. Therefore, it is important that starting volumes among samples are equal to provide consistent results. At the outset of a metabarcoding experiment, or before using beads after a break of several weeks, we recommend “calibrating” the beads (see Support Protocol 2) by cleaning ladder samples with a range of bead concentrations to determine the optimal ratio for retaining target amplicons while removing unwanted artefacts.

The controls should be prepared for sequencing as they can help during the bioinformatics stage to assess quality control thresholds and levels of index hopping. For extraction controls we advise treating them as if they were fecal samples and pooling the replicates together. If the budget permits, positive and negative PCR controls would ideally be tagged and/or indexed and sequenced separately so that patterns of contamination could be tracked for each PCR attempt.

49 Take the prepared Sera‐Mag SpeedBead solution out of the fridge to allow it to come to room temperature. It is critical to vortex until the beads are completely resuspended. There should not be any beads stuck to the bottom or side of the tube, and the solution should appear homogeneous.
50 Make an aliquot of the volume of beads that you will require. The volume of beads to add depends on the expected amplicon size you want to keep and the volume of DNA you want to clean. e.g., If you want to use 0.8× beads to clean ten 15 µl PCR products, then you would add 12 µl beads to each and would therefore need 120 µl beads (you may need to add slightly more for pipetting error).
51 Make fresh 80% ethanol. This must be done each time.
52 Add the appropriate volume of beads to each reaction (e.g., in the example above you would use 12 µl beads) and mix the PCR product and beads thoroughly by pipetting up and down 10 times. The solution should be homogenous after mixing. If you are doing many samples, you may need to mix the prepared bead solution again (e.g., after every 8 samples give the tube of beads a shake).
53 Incubate at room temperature for ≥10 min. During this time get and label new PCR tubes for the elution step.
54 Place the samples on the magnetic plate (e.g., MAGBIO MyMag 96× magnetic plate) to separate. Wait ≥2 min until the solution is clear. The higher the volume of beads the longer this will take to separate.
55 While on the magnet, remove liquid by pipetting and discard without disturbing the beads. If the beads are accidentally disturbed, pipette the liquid back in and wait another ≥2 min for them to separate again.
56 While still on the magnet, add 200 µl of 80% ethanol to each reaction. A reagent reservoir and multichannel pipette can be used here for efficiency.
57 Incubate at room temperature for 30 s.
58 Remove ethanol using a pipette and discard, again being careful not to disturb the beads. Ethanol will be added again in the next step, so it is ok at this point if a small amount is left behind.
59 While still on the magnet, add 200 µl of 80% ethanol to each reaction.
60 Incubate at room temperature for 30 s.
61 Carefully remove ethanol using a 200‐µl pipette and discard.
62 Remove any remaining ethanol using a 10‐µl pipette to get the last drops that might have been missed. Again, be careful not to disturb the beads.
63 While still on the magnet, incubate beads at room temperature for ∼5 min to allow residual ethanol to evaporate.
  Incubation times >5 min can make it difficult to resuspend beads, particularly if eluting in small volumes, but it is also important that the ethanol is completely gone. When dry, the beads may look like little dots or have cracks (like very dry dirt) but should NOT appear wet or glisten at the surface.
64 Take samples off the magnet and add an appropriate amount of 10 mM Tris·HCl, pH 8 (e.g., 15 µl).
  The volume to add depends on the volume you had at the start. The minimum amount for elution is ∼15 µl. If you add more buffer than your starting sample volume, then you will be diluting your amplicon concentration. To concentrate your sample, add less buffer than your starting volume of sample. Pipette up and down 10 times to mix thoroughly. Pipette solution on walls of tube, if necessary, to remove any beads stuck on the side. The solution should appear homogeneous after mixing, but occasionally the beads will appear flaky, and this is ok. During clean‐up we recommend eluting the amplicons in the minimum amount of elution buffer to maximize concentration for subsequent steps.
65 Incubate at room temperature for 10 min OFF the magnet.
66 Place samples back on the magnet plate and incubate for 2 min to allow the beads to separate.
67 Transfer eluent, which now contains your amplicons, to new tubes. Often it is impossible to transfer all of it, and you may need to leave a small amount behind.
68 Store the cleaned products at –20°C.

#### Section 4: Pooling amplicons from multiple primer sets (optional)

If you are using multiple primer sets (e.g., for arthropods and plants), you may pool the purified amplicons prior to indexing to save cost (Fig. 2E). This is only advisable if the primers target different loci (e.g., COI vs rbcL). Avoid pooling primers targeting the same locus, as overlapping regions complicate bioinformatic separation.

69 If pooling loci together quantify the concentration of the purified amplicons for each primer set.
  Amplicons that appear equally bright on an agarose gel can still produce highly unequal read depths during sequencing, with one locus dominating and others underrepresented. To avoid this, we recommend quantifying the concentration of purified amplicons for each locus prior to pooling. Spectrophotometers like the Nanodrop can be unreliable, as absorbance readings are easily skewed by residual contaminants. Fluorescent‐based quantification methods (e.g., Qubit or PicoGreen) offer improved accuracy by binding specifically to double‐stranded DNA, although they can still detect non‐target fragments such as retained primer dimers. More advanced fluorescent‐based instruments (e.g., Bioanalyzer, Qiaxcel, Tapestation) can measure concentrations of specific bands but are more expensive and require substantial equipment investment. Quantitative PCR (qPCR) is arguably the most accurate, as it measures only the concentration of the target locus using fluorescence. However, it can be challenging to implement without appropriate standards of known concentration, and it is more costly due to the need for multiple replicates. Overall, we recommend using a high‐sensitivity fluorescent‐based method if budget allows. Note that fluorescence‐based quantification generally requires the use of optically clear tubes.
70 Calculate the nanomolar concentration of each purified amplicon pool using the equation:
$$\mathrm{nM}\;=\;\frac{\left(\mathrm{ng}/\mu l\;\times \;10^{6}\right)}{\left(660\;\times \;\mathrm{band}\;\mathrm{length}\right)}$$
  where ng/µl is the initial amplicon concentration and band length is the size of the amplicon in base pairs. If multiple bands are present, estimate an average size based on their relative brightness. For example, if a 300 bp band appears to represent ∼90% of an individual sample's overall quantity, but a faint 100 bp band is also visible, you might estimate that the size is: (300 bp  ×  0.9) + (100 bp  ×  0.1) = 280 bp. Using nanomolar concentrations in subsequent steps helps standardize based on number of molecules, preventing over‐representation of shorter amplicons.
71 Amplicon pools for each locus should be combined at equal nanomolar concentrations, diluting more concentrated products to match the lowest. Ideally, aim for concentrations >5 nM to ensure >10 nM post‐indexing. If some samples are very weak (e.g., extraction controls or negative controls) they may still be pooled but will receive proportionally fewer sequence reads since their concentration is lower than other samples.

#### Section 5: Indexing amplicons

In this section, a second PCR with a limited number of cycles extends the adapter overhangs from the locus‐specific PCR to full‐length Illumina adapters. This reaction also incorporates a unique index pair for each sample, enabling pooled sequencing while ensuring reads can be correctly assigned during bioinformatic processing (Fig. 2F).

72 Assign unique indexing primers to each sample and make a record of assigned combinations to be used at the bioinformatics stage.
  We suggest that indices and tags should be used that have a reasonable edit distance (e.g., it requires ≥3 substitutions to change one valid index to another valid index) and that have roughly equal base frequencies at all positions of the index. Glenn et al. (2019) provide a helpful review of the history of indexing and guide to different indexing approaches. Note that tag‐hopping can occur if individually tagged amplicons with different tags are pooled and subsequently amplified together in an indexing PCR.
73 Label a new set of PCR tubes and record information about the location of each sample. This will serve as another method to identify the sample if the label is rubbed off the tube during the PCR.
74 Place the KAPA HiFi HotStart ReadyMix, nuclease‐free water, indexing primers, and PCR products on ice to keep them cool. Mix thoroughly before use but be sure to mix the KAPA HiFi ReadyMix by hand. Centrifuge 30 s at 9240  × *g*, room temperature, to collect the reagents at the bottom of the tube.
75 Add 2.5 µl of each indexing primer to each corresponding empty PCR tube using a new tip for each primer. Each tube should receive a unique set of primers; primers cannot be added to the master mix.
76 Add 10 µl of each amplicon to the assigned tube using a new pipette tip for each sample.
77 Make a master mix of KAPA HiFi HotStart ReadyMix and nuclease‐free water. 25 µl of KAPA HiFi HotStart ReadyMix and 10 µl nuclease‐free water are required for each reaction. Multiply the volume of each reagent required for one reaction by the number of samples to determine the volumes of KAPA HiFi and water needed for the master mix (add 10% for pipetting error, see Table 3).

**Table 3:** KAPA HiFi ReadyMix Master Mix Volumes Required for Indexing

Reagents	Reaction (µl)	Master Mix (µl)
KAPA HiFi ReadyMix	25	25 × *n* samples
H_2_O, nuclease‐free	10	10 × *n* samples

78 Add 35 µl master mix to each tube using a new pipette tip for each tube. Mix the tubes gently.
  It is possible to use smaller PCR reaction volumes, but we have found that using a large reaction and concentrating in subsequent bead cleaning steps consistently gives high yields suitable for sequencing.
79 Place the tubes into the thermocycler and run the required program (see Table 4).
  This program may seem unusual but works well with this polymerase. Cycle number can be adjusted based on product strength, though it is best to keep cycles low (ideally 8 to 12) to minimize errors, chimeras, and bias. Using a high‐fidelity polymerase like KAPA HiFi works well to reduce error rate (Sze & Schloss, 2019).

**Table 4:** Thermocycler Settings for Indexing Using KAPA HiFi ReadyMix

Process	Temperature (°C)	Time (min:s)	
Polymerase activation	95	3:00	
Initial denaturation	98	0:30	
Denaturation	98	0:20	×12 cycles of denaturation, annealing, and extension*^a^*
Annealing	63	0:30	
Extension	72	3:00	
Holding temperature	15	Forever	

^*a*^ It is recommended to keep cycles low (ideally 8‐12) to minimize errors, chimeras, and bias.

80 Perform an agarose gel electrophoresis using 5 µl of each sample to confirm that the amplicon sizes have shifted as expected with the extension of the Illumina overhangs to full length. Each sample will have 45 µl remaining. Store this in the freezer until the next step.
  Technology such as the BioAnalyzer or TapeStation can precisely quantify product sizes, though costs increase if sample numbers are large. Agarose gel electrophoresis can also adequately demonstrate size shifts. We suggest assessing the size of all the samples, as this is also necessary for determining nanomolar concentrations for pooling samples in a later step. In theory, all products should be the same target size, but some may exhibit residual primer dimer or non‐specific amplification, which should also be considered (see Troubleshooting).

#### Section 6: Amplicon purification, normalization, and pooling

In this section, the indexed amplicons, now with full‐length Illumina sequencing adapters, are cleaned again, the concentration of the amplicons is determined, and then samples and loci are combined into a single sequencing pool at equimolar concentrations (Fig. 2G and 2H).

81 Purify the amplicons following the protocol outlined in Section 3 above.
  This step removes residual impurities from the indexing PCR, such as leftover nucleotides and, importantly, excess indexing primers that can increase the risk of index hopping during sequencing. Take extra care to use the correct volume of magnetic beads, as the starting volume is now 45 µl (higher than in the first clean‐up), requiring a proportionally greater volume of beads. The elution volume should remain at 15 µl to help concentrate the final samples.
82 Quantify the cleaned indexed PCR products. We recommend performing a broad range Qubit or other fluorescence‐based kit following the manufacturer's instructions.
83 Calculate the nanomolar concentration of each purified amplicon pool. See Section 4, step 70 for details.
84 Dilute the cleaned indexed PCR products as necessary to equal concentration. For Illumina platforms a minimum concentration of 10 nM is ideal, though lower concentrations (2 nM) may be acceptable. We typically aim for slightly higher (20 nM) as concentrations can sometimes be overestimated with fluorescence‐based kits.
85 Pool all samples in equal nanomolar concentrations and in equal volumes. In practice, the volume used for each sample should be large enough that it can be accurately pipetted (e.g., 2 µl per sample). The required final total volume of the sequencing pool will depend on the sequencing provider and sequencing run but can range from 20 µl to >100 µl.
  An alternative to this labor‐intensive approach is technology that can normalize samples automatically, such as SequalPrep Normalisation Plates. This method is more time efficient and helps minimize pipetting errors. However, the kit has a relatively large up‐front cost and has minimum input DNA requirements that may not be possible for some samples or controls (SequalPrep Normalization Plate Kit, 96‐well, Thermo Fisher Scientific, cat. no. A1051001).

#### Section 7: Sequencing pool validation

Before submitting the pool for sequencing, it is important to verify both its quality and concentration (Fig. 2I).

86 Conduct quality control of the sequencing pool fragment size following the manufacturer's protocol of the desired method.
  Ideally, quality would be assessed using a high‐resolution electrophoresis platform such as an Agilent TapeStation, which provides precise size profiles of the amplicons. Alternatively, agarose gel electrophoresis of the pool can be used. This step can identify an excess of PCR artefacts at sizes other than the size of your target fragment. If excessive amounts of off‐target products (e.g., primer dimer) are identified in the pool then a large proportion of the sequences may be off‐target, or the sequencing run can have reduced quality. A size selection may be necessary to remove these artefacts as they will produce sequencing reads. Size selection could be performed very accurately using technology such as a Pippin Prep (Sage Science), or manually via agarose gel electrophoresis and a standard gel extraction kit. This can result in substantially decreased concentrations and may necessitate a further PCR to boost concentrations to the required level.
87 Assess concentration of sequencing pool following the manufacturer's protocol of the desired method.
  The final concentration of the sequencing pool can be accurately determined by qPCR. When using an Illumina library quantification kit and associated standards, this approach also has the added advantage that it only quantifies molecules that have been successfully constructed into Illumina sequencing libraries with full length adapters. This ensures that only high‐quality pools are sent for sequencing and avoids wasted expense of unsuccessful sequencing runs. If qPCR quantification suggests much lower concentration than expected, it may indicate issues with the indexing PCR. An alternative to qPCR would be to estimate concentration of the final pool using a fluorescence‐based method like Qubit. Many sequencing providers perform qPCR upon receipt of the sequencing pool.
88 Prepare the metadata file containing sample names and the index sequences for the sequencing provider.
  Prior to sequencing, the sequencing provider will require some details. These include information relating to the concentration and volume of the sequencing pool, number of unique index combinations included, their sequences and associated sample names. They will often ask about the sequencing adapters used, which are provided in Figure 1 and are of the Nextera style. Further considerations include requirement for single vs paired end sequences, length of the sequences, and the amount of data required. See Critical Parameters below.

## Support Protocol 1 MAKING A SpeedBead SOLUTION

The size of DNA fragments that the beads will remove depends on the final concentration of PEG. This protocol recommends a concentration of 18% (w/v), which results in a size cut‐off similar to the cut‐off of commercial AMPure beads. You should experimentally infer the size cut‐off using DNA ladders (see Support Protocl 2). Fermentas GeneRuler Ultra Low Range Ladder and NEB ladders work well.

### Materials

PEG‐8000 powder (Promega, cat. no. V3011)
5 M NaCl (Current Protocols, 2006)
1 M Tris·HCl, pH 8.0 (Current Protocols, 2006)
0.5 M EDTA, pH 8.0 (Current Protocols, 2006)
H_2_O, nuclease‐free (New England Biolabs, cat. no. B1500S)
100% Tween‐20 (Sigma‐Aldrich, cat. no. P2287)
Sera‐Mag SpeedBeads, 15 ml, carboxylate‐modified microparticles (Thermo Scientific, cat. no. 6515‐2105‐050250)
TE buffer (10 mM Tris‐HCl, 1 mM EDTA, pH 8.0) (Current Protocols, 2006)

10‐ml serological pipette
200‐ and 1000‐µl pipettes and filter tips
50‐ml centrifuge tube
2‐ml snap‐cap tubes, sterile
Magnetic rack, e.g., MAGBIO MyMag 96× magnetic plate
Aluminium foil

1 Add 9 g PEG‐8000 powder to a 50‐ml centrifuge tube.
2 Add 10 ml of 5 M NaCl (using a 10‐ml serological pipette), 500 µl of 1 M Tris·HCl, and 100 µl of 0.5 M EDTA to the centrifuge tube using 1000‐ and 200‐µl pipettes, respectively. Add nuclease‐free water to about 49 ml. Shake the centrifuge tube until all the PEG has dissolved. Add 27.5 µl Tween‐20 and mix thoroughly.
3 Resuspend the stock solution of Sera‐Mag beads by shaking. The beads should be a homogenous brown colour, with no traces of beads stuck to the bottle.
4 Transfer 1 ml resuspended bead suspension to a 2‐ml tube and place in a magnetic rack to pellet the beads to the side of the tube (the solution will become clear, generally takes ≥2 min). Remove the storage buffer using a pipette.
5 Wash the beads by taking them off the magnet, add 1 ml TE buffer, mix well, then place the beads back on the magnet until the beads move to the side of the tube and the solution becomes clear (≥2 min), and then remove the solution.
6 Repeat step 5.
7 Fully resuspend the beads in 1 ml TE buffer.
8 Add the bead suspension to the centrifuge tube and mix thoroughly. Wrap the centrifuge tube in aluminium foil to protect the beads from the light and store at 4°C.

## Support Protocol 2 CALIBRATING THE SpeedBead SOLUTION

Prior to using the beads to clean your PCR products, they need to be calibrated to determine the correct concentration of the beads to remove artefacts but keep valid PCR products. We recommend doing this when making a new bead solution, and every 2 months.

### Materials

AMPure or home‐made Sera‐Mag SpeedBeads (see Support Protocol 1)
100% ethanol (Sigma‐Aldrich, cat. no. E7023)
H_2_O, nuclease‐free (New England Biolabs, cat. no. B1500S)
10 mM Tris·HCl, pH 8 (Sigma‐Aldrich, cat. no. 93363)
Gel electrophoresis reagents:
  Agarose (Sigma‐Aldrich, cat. no. A9539)
  Ethidium bromide (Thermo Fisher Scientific, cat. no. 15585011)
  Low molecular weight DNA ladder (New England Biolabs, cat. no. N3233L)
  Tris‐borate‐EDTA (TBE) buffer (Thermo Fisher Scientific, cat. no. B52)

0.2‐ml PCR tubes
Vortex mixer
50‐ml centrifuge tube
10‐, 20‐, and 200‐µl pipettes and filter tips
2‐ml snap‐cap tubes, sterile
Magnetic rack, e.g., MAGBIO MyMag 96× magnetic plate
Gel electrophoresis system
Gel imaging system

1 Select the bead concentrations to test. We typically test concentrations from 0.6× to 2.0×.
2 Label 0.2‐ml PCR tubes with your chosen concentrations to test.
3 Take the beads out of the fridge and allow them to come to room temperature. It is critical to vortex until the beads are resuspended. There should not be any beads stuck to the tube, and the solution should appear homogeneous.
4 Make fresh 80% ethanol in a 50‐ml centrifuge tube (it is important to do this each time), including 5 µl extra per sample for pipetting error (e.g. 405 µl per sample). For example,
  a. For 1 sample: 324 µl of 100% ethanol and 81 µl nuclease‐free water.
  b. For a master mix of *n* samples: 324 µl × *n* of 100% ethanol and 81 µl × *n* nuclease‐free water.
5 Prepare the test samples:
  a. Make a solution in a 2‐ml microcentrifuge tube of 2 µl DNA ladder (low molecular weight or 100 bp from NEB work well) and 18 µl nuclease‐free water for the total number of samples plus an extra sample for pipetting error.
  b. For example, 18 µl ladder and 162 µl water for 8 concentrations to test.
  c. Mix this well and aliquot 20 µl of the diluted ladder into each of the PCR tubes.
6 Make an aliquot of the volume of beads needed and add the appropriate volume to each tube. For example:
  a. 0.6× = 20 µl diluted ladder + 12 µl beads.
  b. 0.8× = 20 µl diluted ladder + 16 µl beads.
  c. 1.0× = 20 µl diluted ladder + 20 µl beads.
  d. 1.2× = 20 µl diluted ladder + 24 µl beads.
  e. 1.4× = 20 µl diluted ladder + 28 µl beads.
  f. 1.6× = 20 µl diluted ladder + 32 µl beads.
  g. 1.8× = 20 µl diluted ladder + 36 µl beads.
  h. 2.0× = 20 µl diluted ladder + 40 µl beads.
7 Mix well by pipetting up and down, then incubate at room temperature for ≥10 min. During this time get and label new PCR tubes for the elution step.
8 Place on a magnetic plate to separate. Wait ≥2 min until the solution is clear.
9 While on the magnet, remove liquid using a pipette and discard without disturbing the beads. If the beads are accidentally disturbed, just pipette back in and wait another 2 min for them to separate again.
10 While still on the magnet, add 200 µl of 80% ethanol to each reaction.
11 Incubate at room temperature for 30 s.
12 Remove ethanol using a pipette and discard, again being careful not to disturb the beads. Ethanol will be added again in the next step, so it is ok at this point if some gets left behind.
13 While still on the magnet, add 200 µl of 80% ethanol to each reaction.
14 Incubate at room temperature for 30 s.
15 Carefully remove ethanol using a 200‐µl pipette and discard.
16 Carefully remove ethanol using a 10‐µl pipette to remove any last drops that might have been missed. Again, be careful not to disturb the beads.
17 While still on the magnet, incubate the beads at room temp for ∼5 min to allow residual ethanol to evaporate.
  Incubation times >5 min can make it difficult to resuspend beads, particularly if eluting in small volumes, but it is also important that the ethanol is completely gone. When dry, the beads may look like little dots or have cracks (like dry dirt) but should NOT appear wet or glisten at the surface.
18 Take the samples off the magnet and add 20 µl of 10 mM Tris·HCl pH 8. Pipette up and down 10 times to mix thoroughly. Pipette solution on walls of tube, if necessary, to remove any beads stuck on the side. The solution should appear homogeneous after mixing, but occasionally the beads will appear flaky, and this is ok.
19 Incubate at room temperature for 10 min OFF the magnet.
20 Place the samples back on the magnet plate and incubate for 2 min to allow beads to separate.
21 Transfer eluent to new 0.2‐ml PCR tubes. Often, it is impossible to transfer all of it, and you may need to leave a small amount behind.
22 Run a 2% agarose gel and load 10 µl of the bead‐cleaned products. Also load 10 µl of the remaining uncleaned diluted ladder sample and an undiluted ladder sample to use as comparison. The gel should be run slowly for ∼1 hr at 100 V to allow for good separation of the bands.
23 Take a photograph of the gel using a gel imaging system. You should see a gradient across the gel with the lowest bead concentrations missing the lower ladder bands and the higher bead concentrations retaining everything. If the “gradient” is not perfect, this is likely due to pipetting error (which often happens for people new to bead cleaning).
24 Select the bead concentration according to the size of DNA that you wish to retain.

## Basic Protocol 2 QIIME2 BIOINFORMATICS WORKFLOW FOR METABARCODING DATA

**Figure 3:**
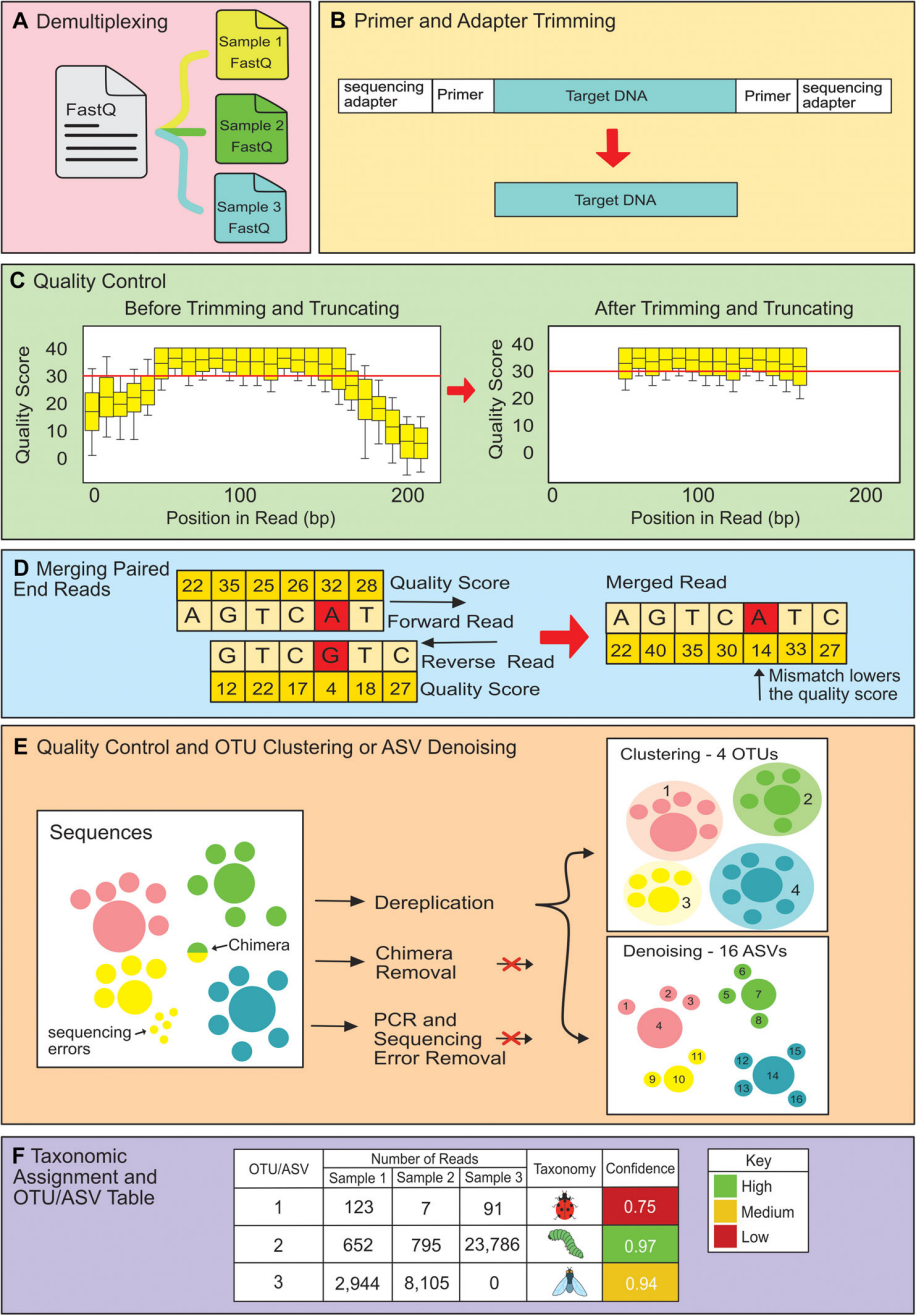
Overview of the bioinformatics process: (**A**) demultiplexing the raw FASTQ data file from the sequencer into separate files according to sample; (**B**) removal of the primers and adapters; (**C**) the reads are trimmed to a quality score of ≥30 and are filtered to remove ambiguous nucleotides and a high number of errors; (**D**) merging paired‐end reads; and (**E**) quality filtering to remove chimeras and PCR and sequencing errors, followed by the dereplication of reads such that all the reads that encode the same sequence are grouped together. A list of unique sequences and their abundance is produced. Operational taxonomic unit (OTU) clustering is used to obtain OTUs or denoising is conducted to produce amplicon sequence variants (ASVs). (**F**) An OTU/ASV table is produced with taxonomy assigned and an indication of confidence.

Bioinformatics involves processing the enormous number of sequences obtained from samples into a table depicting which focal individuals consumed which diet items, along with the taxonomy of those diet items, represented either as an operational taxonomic unit (OTU) or amplicon sequence variant (ASV) table. OTUs are created by grouping reads into clusters that have a sequence identity above a specified threshold (Kozich et al., 2013). ASVs are generated using denoising algorithms that build an error model to retain predicted biological variation and to discard sequencing errors (Callahan et al., 2016). Bioinformatic workflows vary depending on the software used but the process generally involves demultiplexing (i.e., sorting sequences from each sample into separate files), trimming off adapters, trimming off PCR primers and tags, merging of paired‐end reads, quality filtering, the formation of OTUs or ASVs, and taxonomic assignment (Piper et al., 2019) (see Fig. 3). Programs should be selected that are suitable to meet the objectives of the study, the quantity of sequencing data, and the computational resources available. A recent study by Mathon et al. (2021) compares the best bioinformatic programs to use at each step. Packages such as QIIME2 are beneficial for beginners as the installation of a single program, and the plugins contained in the package, are relatively straightforward. Some individuals may prefer to adapt a pre‐existing highly customized pipeline or code their own pipelines, as bioinformatic packages may not be flexible enough to suit their needs.

In this protocol, we discuss an example bioinformatic pipeline using QIIME2 with a sample dataset composed of sequences obtained from metabarcoding bird and bat feces with this lab workflow. The example dataset and metadata file are available in the Figshare data repository (https://doi.org/10.6084/m9.figshare.29816171.v3). The data are composed of 554 raw paired‐end sequence files that were amplified using the Zeale et al. (2011) primers. The sequences are 157 bp in length and originate from 274 individual birds and bats captured in 16 cocoa farms distributed across Cameroon. The metadata file contains information on the predator species, site and date of sample collection (see “faeces_sample_database.xlsx” in Figshare).

### Necessary Resources

#### Hardware

High‐powered computer with >300 GB RAM and internet access

##### Software

Linux‐based environment
Qiime2‐amplicon‐2025.7 (https://library.qiime2.org/quickstart/amplicon)
Miniconda (https://www.anaconda.com/docs/getting‐started/miniconda/main)
RESCRIPt plugin (https://github.com/bokulich‐lab/RESCRIPt)
Microsoft Excel

##### Files

QIIME2 Workflow Script (https://doi.org/10.6084/m9.figshare.29816171.v3); the steps described below correspond to the code in this file
16_farms sequencing dataset (https://doi.org/10.6084/m9.figshare.29816171.v3)
Reference sequences and taxonomy files from NCBI

1 Following the QIIME2 Workflow Script found in the Figshare data repository complete the following steps. Download and install QIIME2 Amplicon, following instructions found at: https://library.qiime2.org/quickstart/amplicon.
  Essentially all bioinformatics work involving high‐throughput sequencing requires working on the command‐line. High‐powered Linux based operating systems such as Ubuntu for Windows (https://ubuntu.com/wsl) will facilitate command line analyses used in this protocol. Ensure that you have a sufficient disk quota to download QIIME2 and store your data. The 16_farms dataset in this protocol is ∼4.8 GB in size.
2 Set up the working environment and change the working directory to the folder containing the data.
  Before importing the data, a suitable working directory should be selected and a backup of the data created. We advise saving output files with suitable names to keep the results organized. As sequence data files are often very large, sequencing facilities typically provide compressed sequencing data files. The most used compression format gives the file a .gz extension. It should not be necessary to decompress the files before starting the analysis.
3 Create a temporary directory.
4 Activate QIIME2.
5 Create the manifest file. This tells the pipeline where your input files are located so the manifest file will be different for every user. The sequence files must have the name format number_R1.fastq.gz or number_R2.fastq.gz for this code to work (1 min).
6 Import the sample sequencing data (5 min).
  Before importing the data, it is essential to determine whether the data is multiplexed or demultiplexed. When the data are multiplexed, all the sequencing data is contained within a file for the forward reads (R1) and a file for the reverse reads (R2) and the sequences are not linked to their original sample source. When the data is demultiplexed, the sequences are stored in separate files corresponding to the sample they originated from. It then needs to be determined what type and format the data is. The “Importing data” tutorial on the QIIME2 web page may be used as a guide to classify data as it provides descriptions of several file types and formats. The 16_farms dataset is in the “PairedEndFastqManifestPhred33V2” format and has the type “SampleData[PairedEndSequencesWithQuality]”. To successfully import this data format a manifest file is required, which contains the file paths of each sample.
7 Use Cutadapt (Martin, 2011) through Qiime2 to remove the Ilumina adapters and the ZBJ primers from the 16_farms fastq files (Fig. 3B, see Troubleshooting) (10 to 15 min).
  Identifying the correct direction and position of adapters and primers can cause some confusion so care should be taken to remove them correctly. This code removes the adapters from the 3’ end and the primers from the 5’ end in the 5’ to 3’ direction. The “p‐discard‐untrimmed” parameter was included to remove untrimmed sequences not amplified by the ZBJ primers, as an extra precaution. The parameters “p‐match‐read‐wildcards” and “p‐match‐adapter‐wildcards” were also used to allow matching of IUPAC wildcards when the primers contain degenerate bases. The –p‐cores 20 parameter runs the code in parallel 20 times to speed up the step while –verbose > cutadapters.txt is used to produce a text file detailing how cut adapt was applied to each sequence file.
8 After trimming off the adapters and primers, check the quality of the reads using demux summarize. The proportion of retained sequences is detailed in the Cutadapt report, and the quality of the sequences can be viewed on the QIIME2 webpage (https://view.qiime2.org/) or using FastQC. The cutZBJ.qzv file should be viewed on the QIIME2 webpage to see the quality of the reads and to select truncation lengths for the next step. See below (5 min).
9 Conduct sequence quality control, the merging of paired‐end reads and denoising to create amplicon sequence variants (ASVs) using DADA2 (Callahan et al., 2016) through Qiime2 (20 min).
  To generate ASVs, DADA2 trims sequences based on read quality and expected amplicon length (Fig. 3). The p‐trim‐len‐f and p‐trim‐len‐r parameters define where sequences are removed from the 5′ end of forward and reverse reads, while p‐trunc‐len‐f and p‐trunc‐len‐r specify the truncation point at the 3′ end, typically where read quality declines. Reads shorter than the specified truncation lengths are discarded (see Troubleshooting). Care must be taken not to over‐truncate, as DADA2 requires a minimum overlap of 20 bp between forward and reverse reads to successfully merge them. This overlap can be calculated by summing the lengths of the truncated forward and reverse reads and subtracting the expected amplicon length. Alternatively, the p‐trunc‐q parameter allows truncation based on a specified quality threshold; we recommend a minimum Phred score = 20. In our case, the ZBJ amplicons had high‐quality scores at the 5′ end, so no trimming was applied there. Instead, we truncated the forward reads to 105 bp and reverse reads to 125 bp, both of which had Phred scores >35. Only 9% of reads were shorter than these thresholds, meaning minimal data loss. Extending the truncation length would have included more bases but resulted in the exclusion of shorter reads. Our chosen lengths provided a 73 bp overlap (105 + 125 − 157), well above DADA2's 20 bp requirement.
  Reads are also filtered based on the number of ambiguous bases and the expected number of errors, which is calculated from the quality scores assuming independent error probabilities at each position (Edgar & Flyvbjerg, 2015). It is also important to ensure primers are properly removed prior to processing, as residual primer sequences can lead to substantial read loss during filtering. Next the filtered reads are dereplicated such that all the reads that encode the same sequence are grouped together and a list of unique sequences and their abundance is produced (Callahan et al., 2016). The data are then denoised by an algorithm developed from a model of the errors that removes potential sequencing errors and divides the sequences further into amplicon sequence variants (ASVs). ASVs are inferred unique reads that have a corresponding abundance and quality profile. Following denoising, DADA2 removes chimeras. These are formed during PCR amplification typically due to incomplete primer extension. During subsequent PCR cycles, the incomplete amplicons act as primers and are extended to form reads that are made up of sequences from two samples (Meyerhans et al., 1990). DADA2 also merges paired end sequences (see Fig. 3D) and creates a feature table that contains the number of times each ASV was found in each sample (Fig. 3F).
  Currently sequence variants are created either by denoising or clustering. In addition to DADA2, Deblur (Amir et al., 2017) also generates models to address potential sequence errors and to produce ASVs. Conversely, clustering programs such as VSEARCH (Rognes et al., 2016) and USEARCH (Edgar, 2010) group sequence variants into molecular operational taxonomic units (OTUs) at some user‐defined similarity, typically 97% to 99% (Liu et al., 2020). The level to cluster sequence data into prey OTUs needs to be carefully selected as it has a strong influence on network analysis results (Clare et al., 2016; Hemprich‐Bennett et al., 2021). Some recent studies suggest denoising methods are advantageous as ASVs produced by denoising detect more unique taxa because they are able to potentially separate taxa differing by only a single nucleotide (Nearing et al., 2018; O'Rourke et al., 2020; Porter & Hajibabaei, 2020). However, one advantage of OTU clustering is that while it may lose some taxonomic information, it prevents the artificial overinflation of taxa by grouping sequences that vary only slightly rather than treating them as unique taxa.
10 Make visualization files of the DADA2 outputs (5 min).
  DADA2 produces three output files. The first file contains a feature table that contains the number of times each ASV was found in each sample. The second file contains stats that summarize how many reads were retained after each filtering step and should be viewed to confirm that denoising was carried out satisfactorily. If the retention number is low an error may have occurred earlier in the pipeline such as during primer removal or the DADA2 parameters may need to be adjusted such as reducing the quality score threshold or altering the truncation length. The third file contains representative sequences and contains a summary of ASV sequence length and an interactive sequence link that allows BLAST searches to be conducted directly from the browser for taxonomic assignment. The average merged read length should be checked that it matches the expected amplicon length. If it is too long primers or adapters may not have been removed successfully.
11 Either skip to step 16 and use the ZBJ COI reference database and classifier developed for this article or follow steps 12 to 15 to produce your own.
12 Install RESCRIPt following instructions found at: https://github.com/bokulich‐lab/RESCRIPt.
13 Download the reference sequences and taxonomy files from NCBI (∼4 hr) by following the code provided in the QIIME2 Workflow script.
  As this is a very large download it is NCBI policy that downloading occurs between 9 p.m. and 5 a.m. Eastern Standard Time unless at weekends.
14 Follow this tutorial to produce a primer specific database: https://forum.qiime2.org/t/using‐rescripts‐extract‐seq‐segments‐to‐extract‐reference‐sequences‐without‐pcr‐primer‐pairs/23618 (Robeson et al., 2021; Rognes et al., 2016).
15 Train the naïve bayes classifier.
  The training process requires ∼44 hr and 300 GB of RAM. Memory usage can be reduced by adjusting the –p‐classify‐chunk‐size parameter and selecting a smaller chunk size than the default. However, this will increase the overall runtime.
16 Assign taxonomy to the ASVs using the naïve bayes classifier created in steps 12 to 15 or the reference database and classifier developed for this article in the Figshare repository (∼1 hr and 140 GB RAM).
  Various strategies exist to taxonomically classify ASVs. For instance, taxonomy can be assigned using alignment software like BLAST (Camacho et al., 2009), other algorithms such as VSEARCH (Rognes et al., 2016) or the naive Bayes classifier used in this example that uses machine learning (Bokulich et al., 2018; Pedregosa et al., 2011). Classification accuracy is influenced by the algorithm used for each approach and further study (e.g., using mock communities) is required to evaluate which practice is best in general (O'Rourke et al., 2020). As the suitability of each classifier differs between studies, the best classifier for your study should be carefully considered.
17 Export the feature table and the 16‐farms‐ncbi‐taxonomy file from QIIME2. Change the name of the taxonomy.tsv to biom‐taxonomy.tsv.
18 Change the header of the biom‐taxonomy.tsv file in a notepad document. The header of the biom‐taxonomy.tsv file needs to be changed from “Feature ID Taxon Confidence” to “#OTUID taxonomy confidence” without the quotation marks and with tabs between the words instead of spaces. The code requires the older terminology OTU (produced by clustering) to be used rather than ASV (which we produced with denoising).
  An OTU table is produced when clustering has been implemented during the bioinformatic analyses, while an ASV table will be produced if denoising has been used. OTU/ASV tables commonly used in the literature contain information on the number of sequences that were observed for each OTU/ASV, the samples the OTUs/ASVs were found in, the taxonomy assigned to each OTU/ASV and the confidence at which it was assigned (Fig. 3F).
19 Add the taxonomy file to the feature table to generate the ASV/OTU table following the Qiime2 Workflow code. Download the outputs, then incorporate the confidence scores from the metadata biom‐taxonomy.tsv file into the ASV/OTU table in Excel. Format the table by removing the first row, splitting the taxonomy into separate columns for each taxonomic level, replacing any blank cells with “NA,” and changing the ASV names in the first column to simpler labels (e.g., 1, 2, 3, etc.). The file should be saved in the csv format for statistical analysis.

## Statistical Analysis

Once data have passed quality control and taxonomic assignment, further statistical analysis will likely be required. We provide sample workflows, including scripts and data files in the Figshare repository. A range of qualitative and quantitative metrics can be used to analyze diet metabarcoding data (Deagle et al., 2019). The simplest approach considers qualitative data in terms of presence or absence of prey groups (an OTU/ASV or a cluster of OTUs/ASVs, i.e., diet items grouped by order) in the diet of individuals. More advanced analyses are then applied, such as network or multivariate analyses, many of which are similar to approaches used in other environmental DNA metabarcoding studies. Several analytical frameworks exist for interpreting dietary data, and the most appropriate choice depends strongly on the research question.

Whether metabarcoding can robustly quantify dietary composition is debated (Deagle et al., 2019; Lamb et al., 2019; Piñol et al., 2015). Various biological and technical factors (e.g., time since consumption, life stage of predator and prey, DNA degradation, and PCR biases) affect read counts and may not reflect prey abundance (Pompanon et al., 2012). Several feeding trials indicate that read counts often diverge from actual diet proportions (e.g., in seals, penguins, deer: Deagle et al., 2010, 2013; Nakahara et al., 2015). Presence/absence data can be used to determine the frequency of occurrence (FOO) as a coarse quantitative measure. Conversely, other studies show correlations between read abundance (RRA) and diet composition (Lamb et al., 2019; Mallott et al., 2018; Neby et al., 2021; Thomas et al., 2014). Correction factors calculated from mock communities (premixed DNA from a variety of taxa in known concentrations) sequence data have been suggested as a potential solution to improve relative abundance estimates (Shelton et al., 2023; Thomas et al., 2014, 2016). A simulation experiment has also shown that the RRA reflected the diet more accurately than occurrence data, which can overrepresent rare diet items, and showed that quantitative analyses can be particularly beneficial when the diet is not very diverse (Deagle et al., 2019). As a result, RRA is gaining acceptance as a coarse (though imperfect) index of abundance that must be interpreted cautiously. Best practice recommends combining qualitative and quantitative metrics (Deagle et al., 2019; Lamb et al., 2019). Here, we provide example code using both metrics to implement basic generalized linear models (GLMs), multivariate approaches, and network analyses. These approaches can be applied independently, but they are not mutually exclusive and often provide a richer ecological interpretation when used together (Cuff et al. 2022; Da Silva et al. 2020; McInnes, Alderman, Lea, et al. 2017; Stapleton et al. 2022).

### Data preparation and GLM analysis

Before undertaking statistical analyses, it is essential to refine and organize the final dataset. The 1.organize&clean_metabarcoding.R script in the Figshare repository outlines one approach for preparing diet data for analysis. First, all non‐target ASVs, such as those from contamination or non‐specific amplification (e.g., bacteria), are removed using the argument keep_class in the R function 1.organize&clean_metabarcoding.R. A full description of the function can be found on Figshare. ASVs are further filtered to remove potential artefacts using the asvs_clean argument in the same function. Here, we apply minimal abundance filtering by discarding all ASVs (assigning them zero reads) if they represent <1% of the total reads within each sample (e.g., Da Silva et al., 2020; Mata et al., 2019). Filtering thresholds are a key consideration in diet metabarcoding and can strongly influence ecological interpretations. Some studies set a threshold by removing ASVs below a certain read count or percentage, but there is no universally accepted approach (Drake et al., 2021). If the threshold is set too low, artefacts and false positives may remain; if it is too high, rare but ecologically important diet items may be excluded (Hänfling et al., 2016). Thresholds can be based on contamination levels in controls and then adjusted depending on study objectives. Our function allows these values to be fine‐tuned by lowering the percentage cut‐offs (e.g., reducing from 1% to 0.1% or 0.01%; Alberdi et al., 2020; Deagle et al., 2019). To evaluate whether the percentage cutoff is suitable, the filtered‐out taxa can be examined to ensure that large read numbers of probable diet items are not removed. Finally, all ASVs that could not be assigned to an order are excluded using the remove_NAorders argument. After running this function, the dataset is ready for downstream analyses and retains all information associated with the fecal samples. Removing OTUs/ASVs that are not assigned to an order will further reduce the total number of ASVs retained. This step should only be applied if detailed taxonomic resolution is necessary to address the study's research objectives (Hemprich‐Bennett et al., 2021).

Once the ASV table is filtered, it can be transformed into a binary matrix with “0” representing absence and “1” representing presence of reads or analyzed quantitatively. From a binary matrix, researchers may calculate frequency of occurrence (FOO) or percentage frequency of occurrence (%FOO), i.e., the proportion of individuals in which a prey item is detected. This metric is most reliable when dietary diversity is low (Deagle et al., 2019). Alternatively, there is some evidence that read counts may act as a proxy for prey abundance in the consumer's diet (Alberdi et al., 2019). In this case, the original filtered matrix can be used quantitatively to calculate relative read abundance (RRA), defined as the number of reads for a prey item divided by the total reads detected in the sample (Deagle et al., 2019).

**Figure 4:**
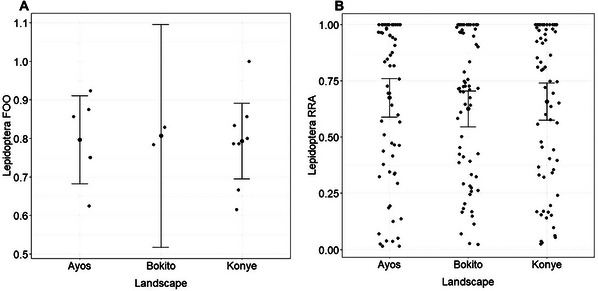
Example generalized linear model results investigating: (**A**) the impact of landscape on Lepidoptera frequency of occurrence (FOO), and (**B**) on Lepidoptera relative read abundance (RRA) in the diets of birds and bats. The plot shows predicted effects with 95% confidence with raw data represented by points.

Finally, the glm_examples.R script can be used to conduct generalized linear model (GLM) analyses on the prepared dataset. The following examples are found in this script. Metabarcoding data can be analyzed using GLMs to answer many ecological questions, for instance, how do animals’ diets vary between habitats (Jarrett et al., 2020), sexes (Da Silva et al., 2020) or sites (McInnes, Alderman, Lea, et al., 2017). In general terms, these factors result in count‐type data that can be described by a discrete probability distribution, such as Poisson or Negative Binomial. For instance, we could compare FOO of one group of individuals to FOO of another group using a Poisson GLM (McInnes, Alderman, Deagle, et al. 2017; Example #1). In some cases, it may be necessary to consider the count data as a proportion; as described above, we may want to consider the proportion of individuals with a certain prey item in their diet (%FOO; Example #2, Fig. 4A). Alternatively, the proportion of ASVs of a certain group (Weighted Abundance; Example #3) or the proportion of reads of a certain group may need to be examined (RRA; Example #4, Fig. 4B). In other words, we want to model our count data as a function of a total number of e.g., individuals (%FOO), ASVs (Weighted Abundance) or reads (RRA). These proportions can be modeled using a Binomial distribution, where the count data become the number of successes, and the total individuals, ASVs or reads the number of trials (Forin‐Wiart et al., 2018; McInnes, Alderman, Deagle, et al., 2017). If using number of reads directly, we would use a count data distribution such as Poisson (Example #5).

### Network analysis

**Figure 5:**
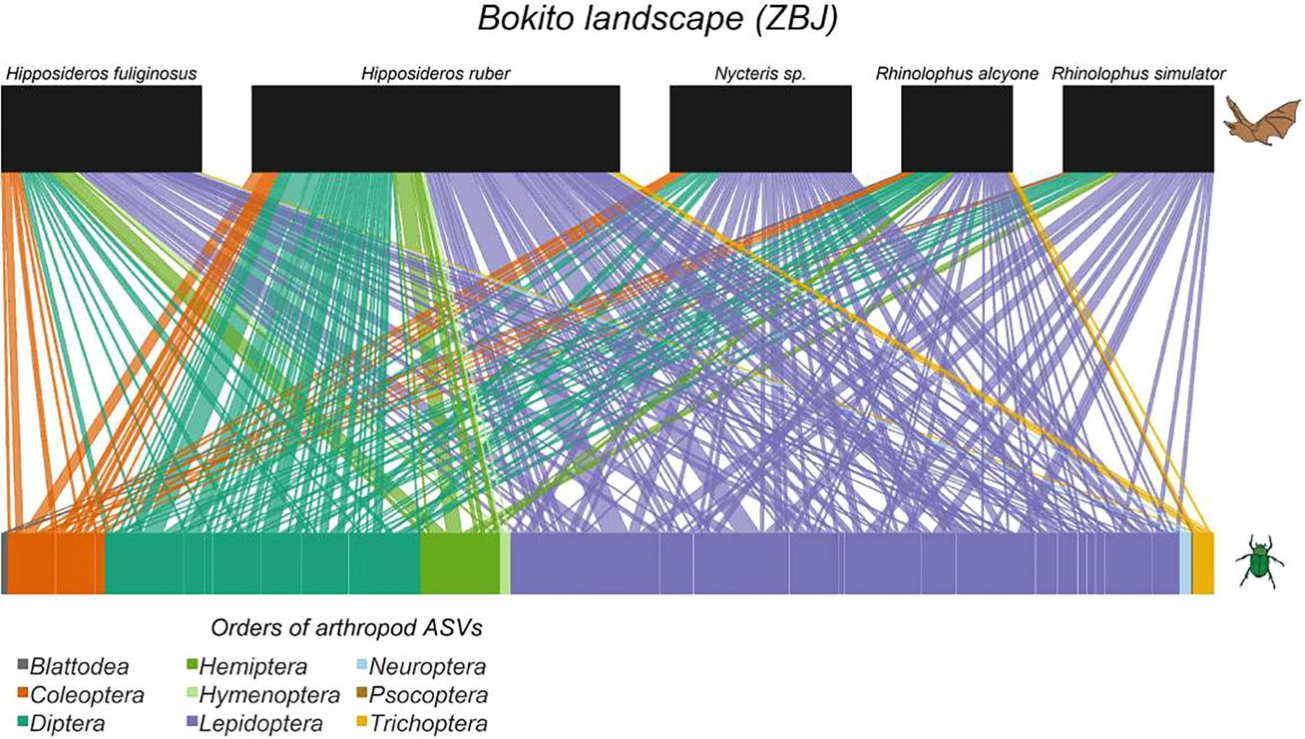
Network analysis investigating a predator–prey network with a higher level represented by predators (bats, top), a lower level by different ASVs (arthropod diet items, bottom) and links between levels by %FOO, with thickness being determined by %FOO. Colors denote different arthropods orders.

**Figure 6:**
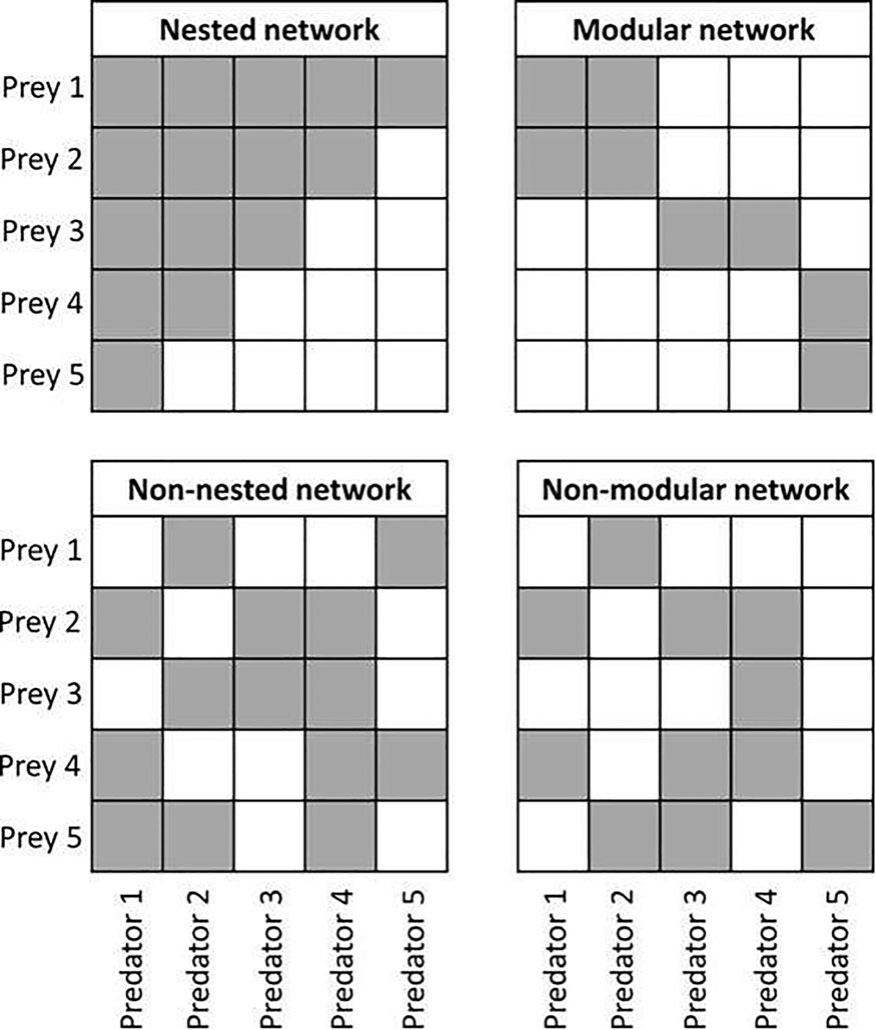
Representation of different network structures based on the interactions between predators and prey. The top row shows networks with high nestedness or modularity, while the bottom row shows networks with low nestedness or modularity.

Networks are another useful data analysis approach for diet metabarcoding data. While ecological networks complement GLMs, they are a more holistic approach to the biotic interactions between species in an ecosystem (Ings et al., 2009). Food‐webs, ecological networks based on the diet of animals, are composed of a top layer with several predator nodes (e.g., bat and bird species) that are connected by links (trophic relationships) to a lower layer with prey OTU/ASV nodes (Fig. 5, Evans et al., 2016). Depending on the research questions, we can build networks where the top layer are individuals, such as the same species but different sex or age groups (Da Silva et al., 2020), species (Hemprich‐Bennett et al., 2021), or other characteristics. Then, we can compare the structure of these networks by computing metrics reflecting species interaction patterns at the network level (e.g., nestedness and modularity) and/or at the species‐level (Delmas et al., 2019; Tylianakis et al., 2010). We provide sample workflow for constructing a network in the building_analysing_networks.R script in the Figshare repository. In our example, we will use modularity and nestedness to investigate how the structure, composition, and resilience of bird and bat species networks vary between three landscapes. Nestedness represents the extent to which the interactions of specialist nodes are nested subsets of the interactions of generalist nodes; in a network with high nestedness, if a specialist species goes extinct, their role within the network will still be maintained by the generalist species (e.g., pollination of a specific plant or consumption of a pest), thus giving the network more stability (Fig. 6). Modularity represents the extent to which network interactions are partitioned into weakly‐coupled modules; a highly modular network may be more resilient as extinction of one species will only influence the other species in the same module, rather than spreading to the whole network (Fig. 6).

In our example we use %FOO to represent our links (Fig. 5), which is a qualitative approach because it does not rely on the number of reads (Deagle et al., 2019). Networks can be very sensitive to sample size and sampling will rarely reflect all species interactions (Hemprich‐Bennett et al., 2021), so we have only included species for which we were able to obtain at least five feces samples from each landscape. Furthermore, we used 1000 null network models (networks with the same number of nodes and links as our original network but with links organized randomly) to account for sampling completeness before comparing networks between landscapes. To determine if the observed value of nestedness and modularity was significantly different from the null‐networks, a *z*‐score can be calculated for these two metrics and each network and group of null‐networks, using the formula “(observed value – mean of the null‐network)/(SD of the null‐network)”. When values are close to 0 it means that the observed value falls within the range expected based on the null‐networks. We constructed and analyzed all networks using the bipartite package (Dormann et al., 2008). See 3.building_analysing_networks.R for a detailed script on how to build and plot networks, evaluate richness and sample coverage, and measure the respective nestedness and modularity.

## REAGENTS AND SOLUTIONS

### Gordon buffer

0.1 M EDTA (ethylenediaminetetraacetic acid) solution (e.g., Promega, cat. no. V4233)
10 mM NaCl (Sigma‐Aldrich, cat. no. S9888)
10 mM of *N*‐lauroylsarcosine sodium salt (Sigma‐Aldrich, cat. no. 61743)
0.1 M Tris·HCl pH 8.0 (Sigma‐Aldrich, cat. no. 93363)

To make 1 L of Gordon buffer, add a small volume of autoclaved deionized water to a sterile bottle. Add 37.22 g EDTA, 0.58 g NaCl, and 2.93 g of *N*‐lauroylsarcosine sodium salt, followed by 100 ml Tris·HCl. Stir gently using a magnetic stirrer to dissolve the dry components, taking care to minimize bubble formation. Add water to bring the volume close to 1 L and then adjust the pH to between 7.5 and 8 using NaCl or NaOH. If necessary, add water so that the final volume is 1L. Expose the solution to UV light for 20 to 30 min (do not autoclave). If bubbles have formed and cannot be avoided, allow the solution to rest overnight before use. This buffer is used during DNA extraction and can be stored up to 1 year at room temperature.

### Longmire buffer

0.1 M EDTA solution (e.g., Promega, cat. no. V4233)
10 mM NaCl (Sigma‐Aldrich, cat. no. S9888)
2% sodium dodecyl sulfate (SDS) (Sigma‐Aldrich, cat. no. L3771)
0.1 M Tris·HCl pH 8.0 (Sigma‐Aldrich, cat. no. 93363)

For 1 L solution combine 500 ml autoclaved deionized water, 100 ml of 1 M Tris·HCl, 200 ml of 0.5 M EDTA pH 8.0, 2 ml of 5 M NaCl, and 20.0 g SDS in this order in a sterilized glass bottle. Add the SDS slowly and stir gently until it has dissolved. Finally bring the solution to 1 L with autoclaved deionized water. This buffer is used for the preservation of fecal samples and can be stored up to 1 year at room temperature.

## COMMENTARY

### Critical Parameters

#### Basic Protocol 1: DNA extraction

Selecting an appropriate DNA extraction protocol for your study is critical. Primarily, column‐based DNA extraction kits are used in the literature (Johnson et al., 2021; Lefort et al., 2020; Shutt et al., 2021; Wray et al., 2021). A number of kits have been developed particularly for fecal samples, which may contain a variety of inhibitors, and manufacturers are constantly developing their kits to increase extraction success (e.g., Qiagen QIAmp Fast DNA Stool Minikit, Norgen Biotek Stool Nucleic Acid Isolation Kit and Zymo Quick DNA Fecal/Soil Kit; see Ando et al., 2020). It is possible to use alternatives to column‐based kits, such as magnetic beads (Jarrett et al., 2020; Thuo et al., 2020; Vo & Jedlicka, 2014) or a cetyltrimethyl ammonium bromide (CTAB)‐based protocol (Oehm et al., 2011), which may prove cheaper. We found the Omega EZNA tissue extraction kit to be effective for dietary DNA from bird and bat fecal samples. However, we recommend selecting a kit that has proven successful in a study system comparable to yours and assessing its performance on surplus samples before use.

If the study species produces large fecal samples, or multiple fecal pellets, some consideration is necessary about how to subsample the feces for DNA extraction. Jedlicka et al. (2017) suggest that random subsamples from bird feces fail to capture the full diet information present in an entire fecal sample. Similarly, while it may be tempting to pool fecal pellets from an individual together prior to extraction, Mata et al. (2019) found this approach to perform poorly in bats compared to results from analyzing each pellet separately, particularly when considering rare diet items. Therefore, it seems a trade‐off exists between exhaustively describing diet for a single individual vs sampling more individuals, and this balance will likely be influenced by budget constraints.

Another critical factor is the amount of feces to utilize for DNA extraction. It may seem intuitive to add all the feces available but doing so can increase the quantity of inhibitors present or overload the process (see Troubleshooting). On the other hand, adding too little feces can result in low DNA yield. The quantity required will also vary greatly depending on the extraction protocol used and the target species. Many studies in the literature do not provide the quantity of feces used for dietary DNA extraction. In this case, if compatible with the extraction kit, we recommend trialing increasing quantities of feces starting with ∼50 mg wet weight. For bats, 60 to 80 mg is often used (Andriollo et al., 2021; Wray et al., 2021); for birds, 50 to 200 mg (Chan et al., 2019; Jarrett et al., 2020; Shutt et al., 2020); for terrestrial mammalian carnivores, 100 to 200 mg is often used (Lu et al., 2021; Massey et al., 2021; Thuo et al., 2019); and for other herbivores, 20 to 60 mg has been used (Christopherson et al., 2019; Goldberg et al., 2020; Kartzinel & Pringle, 2015).

#### Basic Protocol 1: Locus‐specific PCR primer selection

Primer selection is one of the most critical decisions in any metabarcoding study as PCR amplification can introduce unpredictable taxonomic biases (Hsieh et al., 2021). If the aim is to broadly characterize the diets of the focal taxa, such as potentially competing species of subterranean rodents (Lopes et al., 2015) or a trophic network (Sow et al., 2020), then universal primers are recommended. Universal primers are attractive because they can amplify a broad range of taxa, which is particularly valuable for generalist species or diets that are poorly characterized. An ideal locus has conserved primer‐binding regions flanking a variable region to allow discrimination among taxa (Deagle et al., 2014). However, because of these conserved primer‐binding regions they may also co‐amplify non‐diet DNA, including host, microbiome, or parasitic DNA, which can reduce the proportion of usable dietary reads (Lefort et al., 2020). Despite relatively high levels of conservation, primer‐template mismatches still inevitably occur, which can introduce taxonomic biases (Ficetola et al., 2010). Although recent studies have evaluated such biases (Elbrecht et al., 2019; Jusino et al., 2019; Tournayre et al., 2020), predicting their impact on wild animal diets remains challenging. Realistically, highly conserved primer sites may sometimes also flank regions of limited variation, which can hinder taxonomic resolution. Many studies only resolve diet to the family level (Iwanowicz et al., 2016), though genus‐level assignments are sometimes achieved (Goldberg et al., 2020). Using multiple primer sets can mitigate bias and reduce false negatives (Corse et al., 2019; Tournayre et al., 2020), but combining data from different sets is complex and may yield inconsistent results (Kartzinel et al., 2015; Lecaudey et al., 2019).

There are many further considerations for primer selection. When working with feces or other degraded samples, primers that target multicopy loci are particularly advantageous, as they provide more template molecules for PCR. Commonly used targets include mitochondrial DNA, chloroplast DNA, and multicopy nuclear regions, such as ITS and rDNA (Christopherson et al., 2019; Günther et al., 2018; Pitteloud et al., 2021). COI is the most common animal barcode (Elbrecht et al., 2019; Tournayre et al., 2020), with 16S also widely used (Da Silva et al., 2020; Deagle et al., 2014; Shutt et al., 2020). For plants, loci like rbcL and matK are recommended for traditional, single species barcoding (CBOL Plant Working Group, 2009), though for diet analysis, primers targeting short regions of these loci may not provide sufficient genetic variation and hence resolution. Alternatives, such as trnL, trnH‐psbA, and ITS are commonly used (Da Silva et al., 2020; Galimberti et al., 2016; Taberlet et al., 2007; Yang e al., 2016). Amplicon length is also an important consideration. For bird and bat feces, we have found amplification success typically declines above ∼300 to 350 bp, though very short regions can have reduced taxonomic resolution. Fragment length is also an important consideration in relation to sequencing approach. Ideally, amplicons should be short enough that forward and reverse sequence reads can overlap by a minimum of  ~20 bp and be merged during bioinformatics to recover the sequence of the whole fragment (see Critical Parameters: Submitting the library for sequencing). The completeness of reference databases further constrains primer choice. While primers targeting the COI region have been criticized (Deagle et al., 2014), it is still widely utilized because of the extensive reference database available (Hebert et al., 2003).

Omnivores pose a particular challenge for diet metabarcoding, as few primers amplify both plant and animal DNA (Tercel et al., 2021). Researchers must decide whether to focus on a specific diet component or use multiple primer sets (De Barba et al., 2014). Another complication is secondary consumption, which can confound results (e.g., detecting plant DNA from the diet of an herbivorous prey item consumed by an omnivore, which could be confused with direct consumption by the omnivore). Several complementary methods can be used to examine this issue, such as obtaining observational data (Gerwing et al., 2016), conducting morphological analysis of gut contents or feces (Garfinkel et al., 2022; Gil et al., 2020), stable isotope analysis (Bonin et al., 2020), or co‐occurrence analysis (Griffith et al., 2016; Holmes et al., 2019). However, co‐occurrence approaches are exploratory and not definitive proof of trophic links (Blanchet et al., 2020; Da Silva et al., 2020).

Assessing primer suitability is ideally approached through pilot testing (Elbrecht & Leese, 2017; Ficetola et al., 2010). Evaluating candidate primers on potential diet items and fecal samples can identify amplification success and highlight potential biases or inhibitors (see Troubleshooting). Amplification efficiency may vary among host species, likely due to PCR inhibitors associated with diet or physiology. Where resources allow, testing multiple primer sets on a subset of samples before scaling up is the most robust way to ensure chosen primers are appropriate for the study's aims.

#### Basic Protocol 1: PCR optimization

DNA polymerase is an important consideration in PCR, and many are available. Nichols et al. (2018) tested the effects of several polymerases for amplification bias and found that a polymerase's preferred GC content can influence amplification if templates vary widely in GC content. AmpliTaq Gold has been used often in the field of ancient DNA for work with degraded DNA (Grealy et al., 2015; Lendvay et al., 2018; D. C. Murray et al., 2012). High‐fidelity polymerases have lower error rates, though relative read abundance may be impacted (Nichols et al., 2018). We have found that Qiagen Multiplex Taq works well (Jarrett et al., 2020). Qiagen Multiplex Taq was the least biased towards GC content in the tests conducted by Nichols et al. (2018) and effectively retained relative read abundance ratios, though it did produce more errors than other polymerases.

PCR conditions should be carefully optimized for fecal metabarcoding. Temperature is perhaps the main consideration. Often, relatively low annealing temperatures are used (Clarke et al., 2014; Kartzinel et al., 2015; Nboyine et al., 2019; Tournayre et al., 2020), which minimizes stringency and prevents some taxonomic bias; however, low temperatures can allow non‐specific amplification. Since fecal metabarcoding primers often have some sequencing adapter appended to them, any non‐specific products can be sequenced, thus taking away sequences from real diet items. Many PCR machines allow gradient programs to be run where primers can be tested over a range of temperatures simultaneously.

While not an exhaustive list, other factors to optimize in PCR could include denaturing, annealing and extension time lengths, MgCl_2_ concentration, amount of DNA, number of cycles, or potentially inclusion of additives like BSA or DMSO. In general, it is best to use the fewest possible number of PCR cycles while still producing an adequate concentration of amplicons as each cycle of the PCR can generate artefacts. Many diet studies use between 30 and 45 cycles.

To optimize PCR conditions, we recommend starting with some non‐degraded DNA (e.g., DNA isolated directly from a potential diet item itself rather than a fecal sample) as this isolates potential issues (see Troubleshooting). Once primer conditions have been optimized using high quality samples, we suggest testing them on a few surplus or lower priority fecal samples, as amplification success may differ due to degradation and the presence of inhibitors. Since amplification success can be lower with fecal samples, tests may require 7 to 10 samples for accurate assessment.

#### Basic Protocol 1: Amplicon purification approach

There are many clean‐up options available, including, for example, ethanol precipitation, enzymatic treatments (e.g., ExoSAP‐IT), column‐based kits, and magnetic beads. We recommend performing a magnetic bead clean‐up because this approach allows for size selection by adjusting the concentration of beads utilized and can incur minimal costs, though this approach is more time consuming. Enzymatic treatments such as ExoSAP‐IT only degrade single‐stranded DNA primers and excess nucleotides, leaving primer dimers intact. Column‐based kits often retain fragments the size of the primer‐dimer artefacts, e.g., the QIAquick PCR purification kit retains fragments of ∼100 bp or longer (QIAquick PCR Purification Kit, 2023).

#### Basic Protocol 1: Submitting the library for sequencing

Before sending the final pool for sequencing, some additional parameters must be considered: the choice between single‐end and paired‐end sequencing, sequence length, and required sequencing depth. Single‐end sequencing reads from one end of the amplicon, while paired‐end sequencing reads from both ends. Paired end data can allow for overlap and merging of the sequences if the amplicon is shorter than the combined read lengths (e.g., an amplicon of 250 bp sequenced with 2  ×  150 bp reads will recover the full amplicon sequence whereas an amplicon of 400 bp sequence with 2 × 150 bp reads would generate two distinct data sets that cannot be analyzed together). Overlap improves error correction and increases taxonomic resolution (Callahan et al., 2016). Paired‐end sequencing is standard in most fecal metabarcoding studies. In terms of sequence length, longer reads can potentially increase resolution by allowing use of longer regions containing more variable sites, but can increase both sequencing costs and waiting times. Only Illumina's MiSeq and NovaSeq 6000 currently offer reads >150 bp (up to 300 bp and 250 bp, respectively).

Finally, consider the sequencing depth needed, which depends on the study's aims, focal species, dietary diversity, and primers. Common diet items can be identified with relatively few sequences while rare diet items require more. Off‐target sequences should also be considered, either because of artifacts generated during sequencing library preparation (e.g., chimeras, primer dimers) or because of off‐target amplification (predator DNA, fungi/bacterial contamination of sample, etc.). For example, Alberdi et al. (2019) found that ∼25,000 sequences per sample was sufficient to capture most dietary taxa in nine bat species using COI and 16S markers, though OTU discovery continued beyond this. Other systems, especially in biodiverse regions, may require greater depth to detect rare taxa. It is advisable to plan sequencing needs conservatively.

### Troubleshooting

Fecal metabarcoding is a powerful tool for dietary analysis, but its technical and biological limitations must be carefully considered to overcome issues during laboratory processing and bioinformatic and statistical analyses (Table 5; Alberdi et al., 2019). DNA extraction failure is often due to unoptimized protocols, species physiology, or sample quality. Extraction success may be improved by increasing sample quality or preservation at the outset, e.g., by collecting fresher scat samples or using an alternative storage buffer. After sample collection, extraction success can be improved by vigorous sample homogenization (e.g., using a Qiagen TissueLyser rather than simple vortexing) and much longer incubation during lysis steps (≥18 hr). Adjusting the starting weight of feces can also be helpful in dealing with issues like low DNA quantity or the presence of inhibitors. Testing different extraction kits/protocols can also be beneficial, even if time consuming and somewhat expensive at the outset of a project.

**Table 5:** Troubleshooting Guide for Fecal Dietary DNA Metabarcoding

Problem	Possible cause	Solution
DNA extraction failure	Unoptimized extraction method; poor sample preservation	Due to differences in physiology between species, diets, and sampling substrate, DNA extraction protocols should be tested on a case‐by‐case basis
		Extraction success may be improved by using fresher feces samples, an alternative storage buffer, vigorous sample homogenization (e.g., using a Qiagen TissueLyser rather than simple vortexing) and much longer incubation during lysis steps (18 hr or more)
		Increasing starting weight of feces used can also be beneficial if samples have low DNA quantity, whereas decreasing weight can be beneficial if inhibitors are present. We recommend starting with ∼50 mg of feces
Extraction control contamination	Cross contamination from fecal samples; laboratory contamination	To reduce cross‐contamination between extraction rounds, prepare small reagent aliquots and handle sample tubes carefully to avoid touching the inside of the lids
		Conducting DNA extractions in a pre‐PCR or dedicated extraction lab can help, if possible
		Since contamination sources are often difficult to identify, discarding affected reagents and re‐autoclaving tubes is usually the quickest fix
		Complete elimination of contamination is nearly impossible, so sequence extraction controls to account for this during bioinformatics filtering
		See below for general advice on avoiding contamination in the lab
Negative control contamination	Laboratory contamination; cross contamination from DNA samples or positive control	While it may be tempting to handle negative controls with extra caution, they should be treated exactly like your fecal DNA samples to detect any introduced contamination
		To minimize contamination, work within a pre‐PCR hood, use pipettes dedicated solely to your project if possible, discard any compromised PCR reagent aliquots, and reassess your pipetting technique and laboratory environment; additionally, thoroughly clean all laboratory equipment, including sample racks and trays, using appropriate disinfectants such as 20% bleach
		It is nearly impossible to completely avoid contamination during metabarcoding. Sequencing negative control samples is important even if contamination is not detected on an agarose gel; these sequences can potentially be used to guide filtering during the bioinformatics stage
Complete PCR failure including the positive control	User error (e.g., pipetting error, incorrect thermocycler settings) If expe; poor PCR optimization	If the positive control fails, the issue lies with the PCR reaction preparation rather than DNA preservation in the samples
		Verify calculations, thoroughly mix reagents, ensure consistent pipetting and adherence to the protocol, ensure the polymerase activation conditions and annealing temperature are correct
		Further optimization of PCR conditions may be necessary
Positive control amplifies but some or all fecal samples fail	PCR inhibitors; poor PCR optimization	To detect PCR inhibitors, perform a “spike‐in” test by mixing positive control DNA with fecal sample DNA and comparing amplification to the positive control alone via gel electrophoresis. Reduced or failed amplification of the mixed sample indicates inhibitors, therefore further DNA purification or adding less DNA to the PCR reactions may be beneficial
		Quantitative PCR can also be used as a sensitive method for optimizing reactions for maximum amplification
		Further PCR optimization will likely be beneficial
Lower gel bands beneath dietary DNA (∼100 bp)	Primer dimers	Primer dimers occur when the primers bind to each other or themselves
		To improve this, reduce the concentration of primers in the reaction or add more DNA
Upper gel bands above dietary DNA	Non‐specific binding	Optimize the PCR conditions such as increasing the annealing temperature, though this can increase biases in amplification
		It is worth noting, as well, that some primers do naturally produce bands of multiple sizes (e.g., ITS) because the sequences of some species have insertions/deletions
Unexpected dietary results from sequencing data	Primer biases; contamination	If expected diet taxa are missing, primer bias could be an issue. Primer biases can be assessed by testing primers on mock communities containing known quantities of expected dietary taxa and evaluating whether the observed results match the expected composition
		If unexpected diet taxa are present, there may be issues with contamination. See above suggestions for dealing with contamination
Cutadapt failing to remove primers/ adapters	Incorrect primer orientation	In the QIIME2 code, verify the primer orientation by testing both the original and reverse complement sequences, then review the Cutadapt report to see how many reads contain the primer; ensure primers are correctly oriented in the 5′‐3′ direction before proceeding
Poor DADA2 results	Low percentage of samples and reads passing filtering	Reduce the truncation length because if it is too long many shorter reads will be discarded
		Ensure that the reads are trimmed and truncated to a suitable quality score and that the primers and adapters have been removed successfully

PCR failure may arise from multiple factors (Table 5). We recommend in silico testing of primers first to assess primer suitability for candidate diet items, then optimizing PCR conditions (e.g., varying annealing temperature, adjusting DNA concentration, annealing times, etc.) with high quality samples (e.g. expected diet items) to ensure reactions are optimal. Further testing on surplus or low priority fecal samples can be helpful. Once optimized, use of a positive control is essential to isolate potential causes of PCR failure. For example, if the positive control produces amplification, then failure is likely due to sample degradation or the presence of inhibitors. A spike‐in test can be conducted to detect inhibitors. In this test, positive control DNA is added to sample DNA. If amplification fails, then an unknown substance in the sample is inhibiting the reaction. This can be mitigated by additional DNA purification, reducing template DNA, or further optimizing the PCR (e.g., including additives such as BSA). If the positive control fails to produce amplification, then user error, such as pipetting error, missing reagents, incorrect thermocycler settings, or problems with gel electrophoresis (e.g., staining) are options for exploration.

Contamination is one of the largest issues with PCRs (Table 5), and this can be detected through extraction and negative PCR controls. If amplification is detected for an extraction control but not the PCR negative control, the DNA extraction reagent aliquots should be discarded and samples processed again, if possible. If the PCR negative demonstrates successful amplification, PCR reagent aliquots should be discarded and pipetting technique and/or lab environment scrutinized. Thorough cleaning (e.g., with 20% bleach), using sterilized plastics, and filter tips reduce contamination as well. Even successful PCRs may exhibit issues during gel electrophoresis. For instance, small bands (∼100 bp) indicate primer dimers, which can be reduced by lowering primer concentration or adding more DNA, while larger bands suggest non‐specific binding, which may be mitigated by adjusting annealing temperatures.

During bioinformatics many challenges can arise (Table 5). When encountering unexpected problems, we advise scrutinizing error reports, closely re‐reading program manuals, and searching help forums on the internet for advice (e.g., SeqAnswers, Stack Overflow). QIIME provides a user forum and many helpful tutorials. Another useful general strategy is starting from the beginning and checking the input and output of each subsequent step. Sometimes later steps in the bioinformatics can crash because of an undetected problem in a previous step. Consider, for example, if all samples are present, if the number of sequences looks reasonable or if too much data has been lost in a subsequent step. Cutadapt may fail to remove primers or adapters if orientation is incorrect, thus, testing both original and reverse complement sequences and reviewing the Cutadapt report ensures proper trimming. Poor DADA2 results, such as low read retention, are often caused by over‐trimming or truncation, so amplicon length and primer orientation should be carefully checked.

### Understanding Results

Successful implementation of Basic Protocol 1 should yield a high‐quality metabarcoding library from fecal dietary DNA. Libraries should amplify successfully using a qPCR library quantification kit and users should ideally obtain ≥10 nM of pooled library DNA prior to sequencing. This will ensure sufficient coverage for downstream bioinformatic processing. Positive indicators of success throughout this protocol include the presence of correctly sized amplicons after both PCR steps with minimal non‐target binding, as visualized on an agarose gel. Failure to achieve sufficient DNA concentration may indicate suboptimal PCR conditions, degraded template DNA, or issues with bead purification.

Basic Protocol 2 produces an amplicon sequence variant (ASV) table that summarizes the taxonomic identity and read count of dietary items detected in each sample. Each row of this table corresponds to a distinct ASV (diet item), while each column represents an individual fecal sample. Taxonomic assignments include a confidence score that helps determine the reliability of identifications. In our example dataset, 554 sequence files generated an ASV table containing hundreds of unique prey items spanning multiple arthropod orders.

Replicating these protocols with other fecal samples should provide comparable results. Though the read count and taxonomic diversity observed will depend on copious factors, such as the choice of primers, the focal predator species, the range of dietary taxa targeted, the life stage of the predator, and the sequencing platform used.

### Time Considerations

#### Basic Protocol 1

Extracting DNA from one extraction round of 23 samples plus an extraction control typically takes 2 days, though with experience this can be scaled up to two extraction rounds (46 samples, 2 extraction controls) in the same period. For PCR, an efficient strategy is to prepare a first PCR, then while that is thermal cycling, prepare a second. Gel electrophoresis can be conducted for the first PCR while the second PCR is cycling, and so on. Multichannel pipettes can increase efficiency of PCR preparation and loading of agarose gels, though it is still advisable to work in small batches to minimize contamination. Similarly, we advise that the second replicate of any sample set should only be performed once the results of the first PCR are known. This allows for detection of contamination during the extraction process and for DNA volumes to be adjusted if the first replicate yields weak bands. If the first PCR performs well, the second and third PCR replicates can be processed quickly.

Hypothetically, processing three extraction rounds, the maximum that fits on a 96‐well plate (comprising 69 samples, three extraction controls, plus a negative and positive PCR control, totaling 74 samples), would require approximately the following amount of time: 6 days for DNA extractions, 3 days for PCR and gel electrophoresis, 0.5 days for amplicon pooling and 0.5 days for bead cleaning (already optimized). Preparing and assigning indexing primers, performing the indexing PCR, running the associated gels, and bead cleaning the products would take ∼3 days. This would be followed by 1 day to quantify DNA using a fluorescence‐based kit, to calculate nanomolar concentrations based on band length and ng/µL concentration, and for normalization calculations; 1 day to dilute all samples to the same concentration and pool them, and 1 day to validate the library using qPCR and the Agilent TapeStation. In total, this process would take ∼16 working days, equating to roughly 1 month of lab time. If you are using two primer sets instead of one, this will add at least 6 additional days to the workflow. The success of this timescale depends on being thoroughly organized, with all equipment, reagents, and primers prepared in advance, PCR reactions fully optimized, and no major delays. However, given the complexity of the protocol, it is realistic to anticipate setbacks. Therefore, please factor in at least 1 to 2 extra weeks for troubleshooting and unexpected issues.

#### Basic Protocol 2

If you are using the prepared reference database and classifier provided in the Figshare repository, the QIIME2 pipeline should take just over 2 hr to process the samples. However, if you intend to download reference sequences from NCBI and build your own database and classifier, expect the process to take at least 2 to 3 days. The total runtime depends on several factors, including the amount of RAM available, the number of cores allocated, the size of the reference database, the choice of primers, and the number of samples being processed.

### Author Contributions

**Rachel McConnell**: Conceptualization; formal analysis; methodology; software; writing—original draft; writing—review and editing. **Crinan Jarrett**: Data curation; formal analysis; methodology; software; writing—original draft; writing—review and editing. **Diogo Ferreira**: Data curation; formal analysis; methodology; software; writing—original draft; writing—review and editing. **Luke Powell**: Data curation; formal analysis; funding acquisition; writing—original draft; writing—review and editing. **Alma Quiñones**: Investigation; methodology; validation; writing—original draft. **Davide Dominoni**: Conceptualization; supervision; writing—original draft; writing—review and editing. **Andreanna Welch**: Conceptualization; data curation; funding acquisition; investigation; methodology; project administration; supervision; writing—original draft; writing—review and editing.

### Conflict of Interest

The authors declare no financial interest in the opinions or materials presented in this manuscript.

## Data Availability

Complete details including additional information, scripts, and data files used in this manuscript are available in the Figshare repository at: https://doi.org/10.6084/m9.figshare.29816171.v3.
